# Isolation of two triterpenoids from *Phlomis purpurea*, one of them with anti-oomycete activity against *Phytophthora cinnamomi*, and insights into its biosynthetic pathway

**DOI:** 10.3389/fpls.2023.1180808

**Published:** 2023-08-25

**Authors:** L. Fernández-Calleja, M. García-Domínguez, B. Isabel Redondo, J. L. Gómez Martín, C. J. Villar, F. Lombó

**Affiliations:** ^1^ Research Unit “Biotechnology in Nutraceuticals and Bioactive Compounds-BIONUC”, Departamento de Biología Funcional, Área de Microbiología, Universidad de Oviedo, Oviedo, Spain; ^2^ Instituto Universitario de Oncología del Principado de Asturias, Oviedo, Spain; ^3^ Instituto de Investigación Sanitaria del Principado de Asturias, Oviedo, Spain; ^4^ Department Animal Science, Faculty of Veterinary Medicine, Universidad Complutense de Madrid, Madrid, Spain; ^5^ Research and Development Department, Campojerez SL, Jerez de los Caballeros, Badajoz, Spain

**Keywords:** anti-oomycete, root rot, natural compound, oomycete, dieback

## Abstract

*Phytophthora cinnamomi* is an important plant pathogen responsible for dieback diseases in plant genera including *Quercus*, *Fagus*, *Castanea*, *Eucalyptus*, and *Pinus*, among others, all over the world*. P. cinnamomi* infection exerts tremendous ecological and economic losses. Several strategies have been developed to combat this pathogenic oomycete, including the search for novel anti-oomycete compounds. In this work, a Mediterranean vascular plant, *Phlomis purpurea*, has been screened for secondary bioactivity against this pathogen. The genus *Phlomis* includes a group of herbaceous plants and shrubs described as producers of many different bioactive compounds, including several triterpenoids. Triterpenoids are well-known molecules synthesized by plants and microorganisms with potent antioxidant, antitumoral, and antimicrobial activities. We have isolated by HPLC-DAD and characterized by HPLC-MS and NMR two nortriterpenoid compounds (phlomispentaol A and phlomispurtetraolone) from the root extracts of *P. purpurea.* One of them (phlomispentaol A) is active against the plant pathogenic oomycete *P. cinnamomi* (based on *in vitro* inhibition bioassays). Based on their chemical structure and their relationship to other plant triterpenoids, oleanolic acid is proposed to be the common precursor for these molecules. The anti-oomycete activity shown by phlomispentaol A represents a promising alternative to counteract the worldwide-scale damage caused to forest ecosystems by this pathogen.

## 1 Introduction

**Figure 1:**
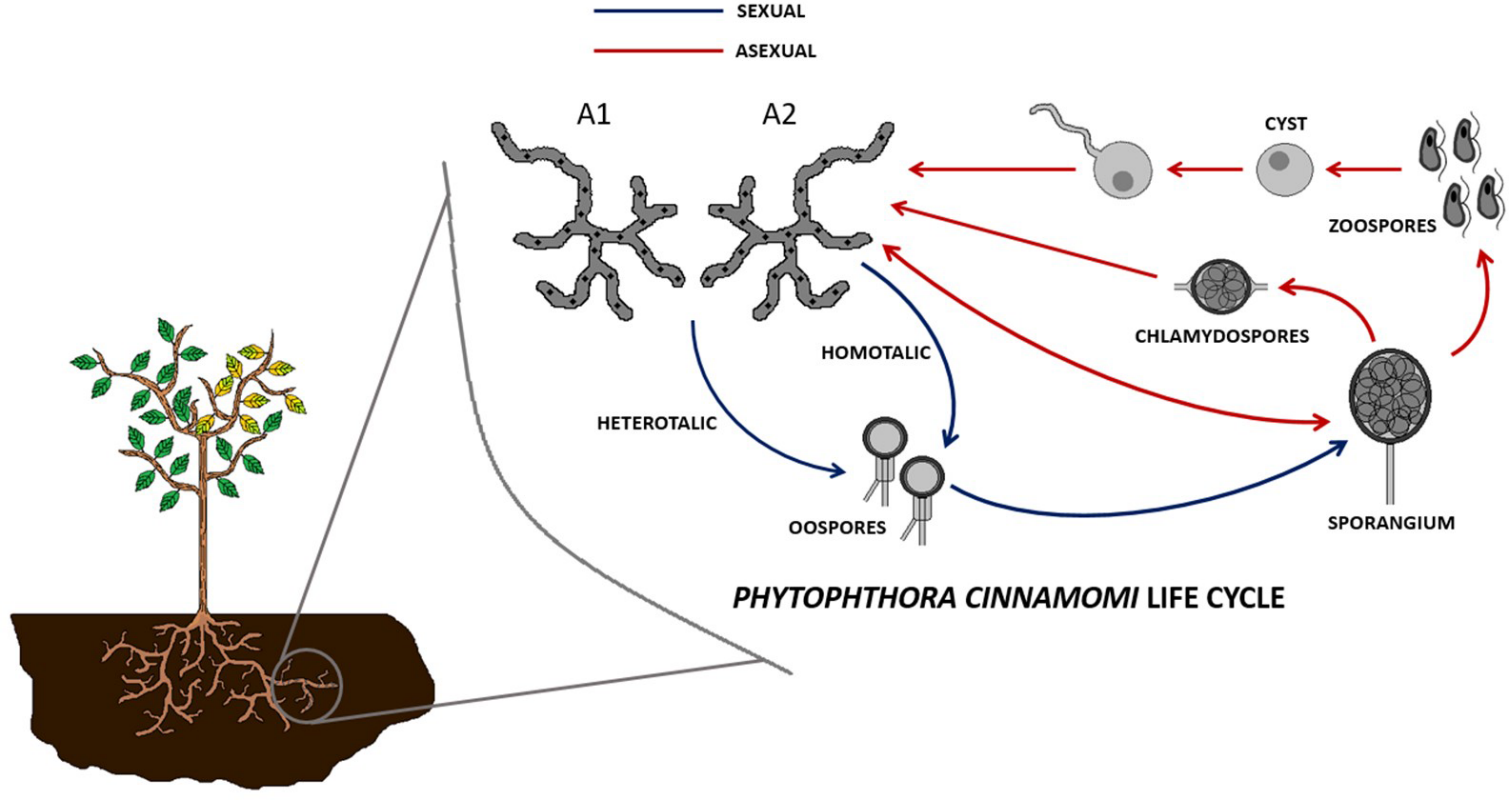
Sexual (via oospores and sporangia) and asexual (via sporangia) cell cycles of *Phytophthora cinnamomi*.

Dieback (also known as root rot) is a plant disease mostly caused by the phytopathogen mold *Phytophthora cinnamomi*, commonly affecting tree species from the genus *Quercus*, such as the cork oak (*Quercus suber*) and the holm oak (*Quercus ilex*), among many other plant genera (*Castanea*, *Eucalyptus*, *Persea*, etc.) (de Sampaio e Paiva Camilo-Alves et al., 2013; Davison, 2018; Hardham and Blackman, 2018). It starts with an infection of the roots, which ends up rotting, followed by yellowing and falling of the leaves, abortion of the fruits, and finally the death of the tree (Hardham and Blackman, 2018). The life cycle of this pathogen has sexual and asexual phases, and it starts with the zoospore, a motile spore that attaches to the elongation part of the host plant root. The attached zoospore secretes biofilm components and becomes a cyst, which germinates, producing hyphae that can grow inter- or intracellularly and secretes enzymes. These hyphae are produced in a heterothallic manner, known as type A1 or type A2. New zoospore-generating sporangia can be generated sexually by mating of A1 and A2 hyphae and formation of oospores or asexually, directly from A1 or A2 hyphae. The enzymes produced by these hyphae degrade the wall of plant cells, causing necrosis and disrupting water uptake during the necrotrophic phase. Alternatively, the pathogen can grow in a biotrophic phase, causing no disease symptoms (**Figure 1**) (Crone et al., 2013; De Andrade et al, 2020).

This disease is a global epidemic, and it is possible to find severely affected ecosystems in North America, Europe, Africa, and Oceania (Burgess et al., 2017). This plague has been affecting more than 60% of nurseries of avocado trees in California over the years, causing up to $40 million in annual losses (Belisle et al., 2019). In Colombia, 30% of nurseries of the same tree have registered infections (Álvarez et al., 2023). By 2015, only 0.08% of Great Otway National Park in south-eastern Australia was not affected by *P. cinnamomi* infestation (Wilson et al., 2020). European chestnut is among the most impacted species in Portugal, where a 52, 725-ton decrease in chestnut production has suffered since 1961 (Fernandes et al., 2021). Originally from Asia, *P. cinnamomi* is responsible for the extensive destruction of chestnut, European oak, holm oak, and cork oak forests that has been observed during the last decades of the twentieth century throughout the entire Mediterranean basin, Western Europe, the Balkans, and the Caucasus, but also throughout the twenty-first century, where mortality persists (Brasier and Scott, 1994; Balci and Halmschlager, 2003; Tziros and Diamandis, 2014; Sena et al., 2018). It is also responsible for the destruction of other forest ecosystems, such as the eucalyptus woods of Western Australia (Arentz, 2017; Davison, 2018; Hardham and Blackman, 2018).

Currently, there is no viable and authorized treatment that serves to effectively eliminate root rot or prevent its spread on a large scale without collateral damage. Control measures are based mainly on inhibitors (such as phosphites) and some divalent cations (such as calcium or magnesium) (Hardham and Blackman, 2018; Ramírez-Gil and Morales-Osorio, 2020). Examples of current treatments for this disease include the use of strobilurin pyraclostrobin (23.3%) (BASF) or the phenylamide mefenoxam (22%) (Syngenta), as well as host plant defense inducers like aluminum tris (*O*-ethyl-phosphanate) (60%) (Bayer) (Neupane et al., 2022). Long-term phosphite treatment is proposed as a method to control *P. cinnamomi* infestation when lacking other options (Barrett and Rathbone, 2018), but there is recent evidence of phosphite-tolerant *P. cinnamomi* isolates obtained from New Zealand (Hunter et al., 2023).

However, there is observational evidence that some herbaceous plants can provide protection against this oomycete to other neighboring plants. One of these herbaceous species is *P. purpurea*, a 1- m-tall shrub that belongs to the *Lamiaceae* family and is endemic to Mediterranean ecosystems. This plant is well adapted to the characteristically arid ecosystem and has been described as a producer of other triterpenoid-type compounds endowed with antimicrobial activity, as well as other compounds with anti-inflammatory (useful for example against ulcerative colitis) and antitumor activities (Álvarez et al., 2012; Algieri et al., 2013; Algieri et al., 2014; Neves et al., 2014; Mateus et al., 2016). In this work, we describe the extraction, purification, and characterization by NMR of two new compounds from *P. purpurea* root samples, one of which has anti-oomycete activity against *P. cinnamomi*.

Terpenes are a wide family of organic compounds formed by several isoprene subunits that generate molecules with a specific number of carbons. They are classified according to that number: monoterpenes (C10), sesquiterpenes (C15), diterpenes (C20), sesterterpenes (C25), triterpenes (C30), and tetraterpenes (C40). Also, regarding the nomenclature of these compounds, it is necessary to make a distinction between triterpenes and triterpenoids. Triterpenes are naturally occurring terpene compounds, whereas triterpenoids are terpene derivatives containing keto or hydroxyl groups (or any other heteroatom) of natural or synthetic origin (Muffler et al., 2011). Triterpenoids can be classified as nor-triterpenoids when lacking one or more methyl groups from the basic molecular structure (Camargo et al., 2022). In our case, the two compounds isolated from *P. purpurea* root extracts, phlomispentaol A (with anti-oomycete activity) and phlomispurtetraolone (first described in this article), possess a 28-norolean spirocyclic triterpenoid skeleton, pointing out the absence of the C28 methyl group as in the oleanane-type basic structure (Camargo et al., 2022). In the present work, we suggest that oleanolic acid is the common precursor for both nortriterpenoids isolated from *P. purpurea* root extracts and we propose the use of phlomispentaol A as an alternative to current control methods against the phytopathogen *P. cinnamomi*.

## 2 Materials and methods

### 2.1 Processing of plant root samples

The samples, consisting of dry root fragments of *Phlomis purpurea* (collected during the summer season), were provided by the collaborating company Campojerez SL, located in Jerez de los Caballeros (38.3212 N, 6.7740 W, Badajoz, South-Western Spain), from plants cultivated in nurseries under the environmental conditions of this area: annual average temperature: 17.1°C; maximal annual average temperature: 23.8°C; minimal annual average temperature: 10. 3°C; average annual precipitations: 447 mm; and average annual number of daylight hours: 2,860 (https://www.aemet.es). All roots were taken from 1- year-old plants.

The root fragments were peeled with a scalpel to obtain their bark. The dry bark, which was fragile and brittle, was micronized in an Ultra-turrax (Sigma Aldrich, Madrid, Spain) process until a fine and homogeneous powder was obtained. The powder was stored at −20°C until its extraction.

The solvent used for extracting the micronized root mass was 99% ethanol (VWR, Barcelona, Spain). In a Falcon tube, the dry root powder of *P. purpurea* was mixed with 99% ethanol in a proportion of 0.1 g/ml. The mixture was shaken for 1 h and centrifuged for 5 min at 9,000 rpm (centrifuge 5804 R, Eppendorf, Madrid, Spain) at 4° C. The ethanolic extract was separated and saved. This extraction process was repeated twice, and then both ethanolic extracts were pooled. In total, 60 g of root samples were extracted. Ethanol was removed from the extract by evaporation under vacuum (IKA HB10, VWR, Spain), and the resulting solid was dissolved in 10 ml of 20% methanol in water. The total process took 3 days to complete.

### 2.2 Solid- phase extraction fractionations

The dissolved root extract was submitted to solid-phase extraction (SPE) fractionation using a Phenomenex Strata C18-E column (55 µm, 70 Å, 10 g, 60 ml) (Phenomenex, Madrid, Spain). The SPE chromatography was performed with methanol and water as buffers A and B, respectively. First, the column was conditioned with 50 ml of 100% methanol, followed by 20% methanol in water. The root extract was then loaded onto the column and eluted in ten 50-ml fractions (C1–C10). C1–C9 fractions consisted of eluates with progressively increasing percentages of methanol (20%, 30%, 40%, 50%, 60%, 70%, 80%, 90%, and 100%, respectively), while C10 fraction was collected with 100% acetone. The fractions were then tested in bioassays, and those that showed inhibitory action against the pathogen *Phytophthora cinnamomi* were selected. The solvents were then removed by evaporation under vacuum and then lyophilized until dry and stored at − 20°C.

### 2.3 High- performance liquid chromatography experiments

After the SPE fractionation experiments, the anti-oomycete molecules were further purified by high-performance liquid chromatography (HPLC)-DAD using an Agilent 1260 Infinity equipment (Agilent, Spain) with a Teknochroma Mediterranea Sea18 column (25 cm × 1 cm, 5 μm particle) (Phenomenex, Madrid, Spain). The active fractions were dissolved in 850 μl of 20% methanol in water and injected for fractionation using four sequential HPLC programs. All gradients were made with analytical-grade solvent B (methanol, 100% (VWR, Spain) or acetonitrile, 100% (VWR, Spain)) and water as solvent A. All solvents contained 0.1% formic acid. HPLC Program #1 (4 ml/min flow rate, B: methanol) was as follows: 0–37 min (73% B), 37–40 min (73%–100% B), 40–41 min (100% B), 41–42 min (100%–10% B), and 42–48 min (10% B). Fractions (each one collected every 0.5 min) were collected from 0 to 47 min. HPLC Program #2 (1.63 ml/min flow rate, B: methanol) was as follows: 0–5 min (50% B), 5–56 min (50%–83% B), 56–70 min (83%–90% B), 70–83 min (90%–100% B), 90–91 min (100%–50% B), and 91–96 min (50% B). Fractions were collected from 36 to 83 min. HPLC Program #3 (1.63 ml/min flow rate, B: methanol) was as follows: 0–27 min (85% B), 27–69 min (85%–92% B), 69–83 min (92%–100% B), 83–85 min (100% B), 85–86 min (100%–85% B), and 86–88 min (85% B). Fractions were collected from 0 to 34 min. HPLC Program #4 (4 ml/min flow rate, B: acetonitrile) was as follows: 0–5 min (10% B), 5–30 min (10%–51% B), 30–55 min (51%–81% B), 55–70 min (81%–100% B), 70–80 min (100% B), 80–82 min (100%–10% B), and 82–84 min (10% B). Fractions were collected from 20 to 67 min. The HPLC programs were selected by estimating the solvent percentage for the elution of the anti-oomycete compound and changing the main slope of the previous program according to the quality of peak resolution.

Between each fractionation experiment, all the fractions were tested in a bioassay against *P. cinnamomi*, and the inhibitory refractions were collected, pooled, dried, dissolved in 20% methanol in water, and reinjected into the next HPLC fractionation program.

Also, after each fractionation round, the positive fractions in the bioassay were analyzed by HPLC-HRESIMS to find out the exact mass of the compounds of interest. The equipment used for these experiments was a Bruker Impact II UHPLC-MS-QTOF (Bruker, Madrid, Spain) with a Zorbax Eclipse Plus C18 column (50 mm × 2.1 mm, 1.8 μm particle size) (Agilent, Spain), and the program (0.25 ml/min) used a gradient of water (solvent A) and acetonitrile (solvent B), both with 0.1% formic acid: 0 min at 10% acetonitrile, 1 min at 10% acetonitrile, 4 min at 35% acetonitrile, 5 min at 35% acetonitrile, 8 min at 100% acetonitrile, 10 min at 100% acetonitrile, 11 min at 10% acetonitrile, and 15 min at 10% acetonitrile.

### 2.4 Antimicrobial activity bioassays against *Phytophthora cinnamomi*

The different fractions obtained from the HPLC-DAD repurification cycles were analyzed using a bioassay against *P. cinnamomi* on Sabouraud Dextrose Agar (SA) solid medium (VWR, Spain). In total, 2,000 μl of the HPLC fractions obtained were evaporated under vacuum, and the solid residue was resuspended in 900 μl of 20% methanol in water. In order to avoid masking the results due to the potential toxicity of the solvent used to resuspend the samples (20% methanol in water), 90 μl of this volume was then added to 4 ml of SA without exceeding a final solvent concentration of 2.25% (v/v) methanol. The SA culture medium was melted and allowed to cool to 50° C before adding the respective fraction to it. The medium was then poured into two 25 -well Petri dishes (4 ml of SA medium per well) (4 cm^2^ each well, Thermo Scientific, Madrid, Spain), making duplicates for each bioassay. Subsequently, each agar well was inoculated with *P. cinnamomi* mycelium extracted from a grown plate using a wide-mouth pipette tip as a punch. In all the bioassays, a blank control was used, containing only the culture medium, as well as a solvent control of the culture medium with 2.25% methanol (used to rule out the possible toxic effect of the solvent on *P. cinnamomi* growth). Mycelium areas were measured by taking a picture of the mycelium, including an object of known size, and analyzing it with an image processing software (Image J, NIH, Bethesda, Maryland, USA), using the size known object as a reference for the measurement. A result was considered positive when a clear reduction of the sample mycelium area was observed compared with that of the blank control in the Image J pictures among assay samples in the microtiter wells. The minimum inhibitory concentration (MIC) experiments were carried out by a liquid bioassay performed in a sterile microtiter plaque. The sample E7F4, containing compound A (phlomispentaol A), was used to determine the MIC against *P. cinnamomi*. An eight-point serial dilution of the sample was elaborated from 150 μg/ml to 2.34 μg/ml, including a well with no sample (0 μg/ml). Another eight-point serial dilution was made as a solvent control from 3.75% to 0.06% methanol concentrations. The total volume for each well was 100 μl. Dilutions were made in an SA liquid culture medium.

### 2.5 Nuclear magnetic resonance studies

Nuclear magnetic resonance (NMR) analyses were performed with a Bruker AVIII-800 spectrometer equipped with a TCI cryoprobe at 800.13 MHz for ^1^H and at 200.20 MHz for ^13^C (Bruker, Spain). Two different HPLC fractions were analyzed (E7F4 and E5E6), in both cases dissolved in 600 μl MeOD.

The following tests were performed on HPLC sample E7F4: ^1^H NMR, ^1^H NMR with presaturation, ^1^H-^1^H COSY, ^1^H-^1^H TOCSY (80 ms), ^1^H-^1^H ROESY (300 ms), ^1^H-^13^C-HSQC, ^1^H-^13^C-HSQC_TOCSY (60 ms), ^1^H-^13^C-HMBC, and ^13^C{^1^H}. The following tests were performed on sample E5E6: ^1^H NMR with presaturation, ^1^H-^1^H COSY, ^1^H-^1^H TOCSY (80 ms), ^1^H-^1^H NOSY (500 ms), ^1^H-^13^C-HSQC, ^1^H-^13^C-HSQC_TOCSY (60 ms), and ^1^H-^13^C-HMBC.

### 2.6 Statistical analysis

The statistical analysis for the bioassay results was made with GraphPad Prism 9.0.2 software. The results were analyzed by a one-way ANOVA comparing all the samples with the blank control and assuming a normal distribution of data. Statistically significant results were considered when obtaining *p*-values below 0.05. Asterisks in the graphics show the level of significance. The regression analysis was carried out using the Microsoft Office Excel program.

## 3 Results

### 3.1 SPE and HPLC-DAD fractionation experiments

**Figure 2:**
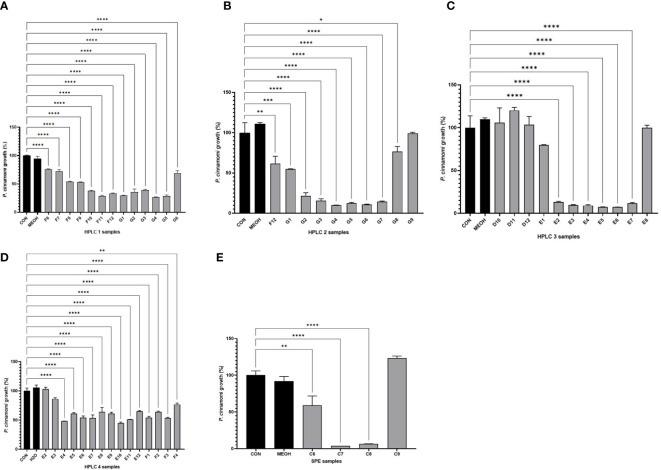
Growth values obtained after bioassays of *Phytophthora cinnamomi* growing on SA medium in the presence of different HPLC rounds or SPE fractions from the *Phlomis purpurea* root extract. **(A)** Percentage of *P. cinnamomi* growth in the bioassay using the HPLC program #1 fractions. **(B)** Percentage of *P. cinnamomi* growth in the bioassay using the HPLC program #2 fractions. **(C)** Percentage of *P. cinnamomi* growth in the bioassay using the HPLC program #3 fractions. **(D)** Percentage of *P. cinnamomi* growth in the bioassay using the HPLC program #4 fractions. **(E)** Bioassay using the SPE fractions. Color legends: the first black column represents the negative control, the second black column represents the negative solvent control, and the gray columns represent the experimental samples (HPLC fractions). *p-value < 0.05, **p-value < 0.005, ***p-value < 0.0005 — statistically significant differences among samples.

The HPLC purification process of the anti-oomycete compounds from the root extracts was monitored using bioassays-guided HPLC fractionation (**Figures 2A–E**). The ethanolic root extract from Phlomis purpurea was initially processed using SPE fractionation, and from the 10 SPE fractions obtained, the bioassays against Phytophthora cinnamomi showed anti-oomycete activity in the cases of fractions C7 and C8 (**Figure 2E**). Also, there were traces of bioactivity in flanking fraction C6, but not in flanking fraction C9.

These C7 and C8 SPE fractions were dried and used for sequential refractionations by HPLC-DAD using the four different programs described in the Materials and Methods section. After fractionation of the samples using HPLC program #1, of the 96 fractions generated, the 10 fractions F8–G5 showed anti-oomycete activity (**Figure 2A**). These 10 fractions, F8–G5, were pooled, evaporated under vacuum, and dissolved in methanol for their fractionation using HPLC program #2. These second-round 96 fractions showed anti-oomycete bioactivity in G1–G7 fractions (**Figure 2B**). These seven fractions were pooled, evaporated under vacuum, and then dissolved in methanol to be processed using HPLC program #3. The 96 fractions from this third HPLC round indicated that fractions E2–E7 maintained the anti-oomycete activity (**Figure 2C**). These five fractions were collected from each HPLC #3 round, pooled, evaporated under vacuum, and then dissolved in methanol to be processed using HPLC program #4. In this last case, fractions E3–F4 maintained the anti-oomycete activity (**Figure 2D**).

**Figure 3:**
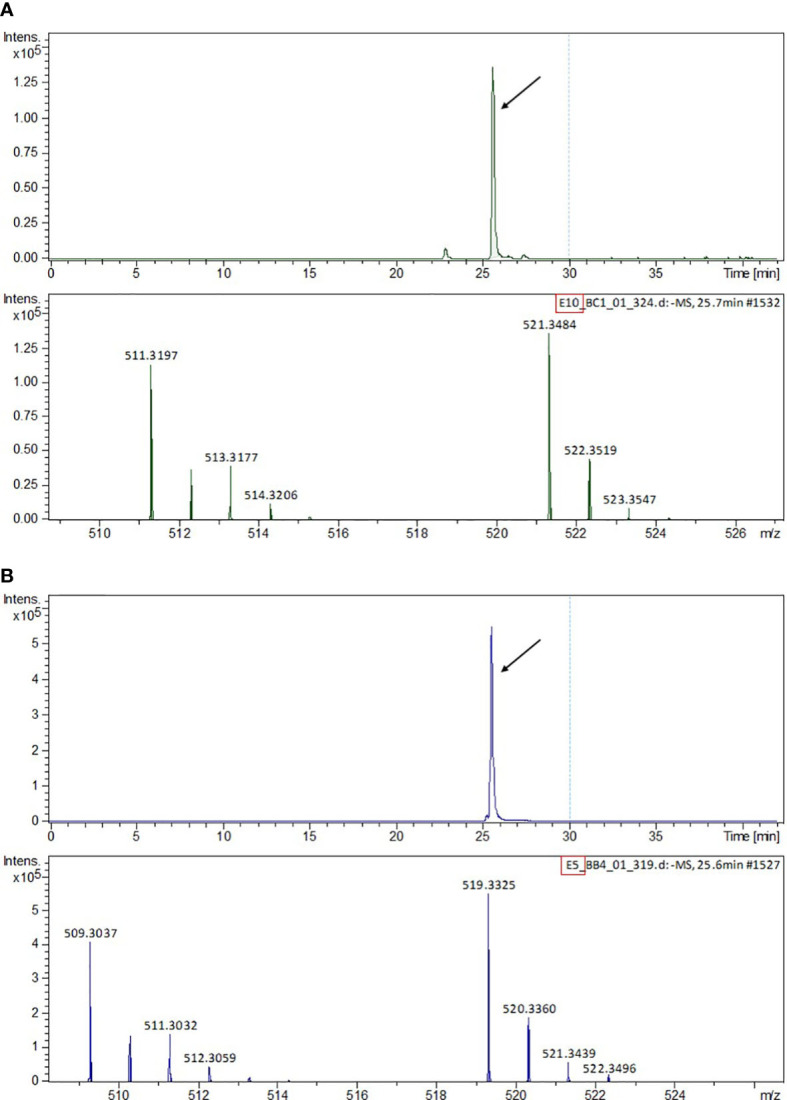
HPLC-MS chromatogram and MS spectra of the m/z ions detected in the pooled HPLC program #4 fractions. **(A)** Fractions E7–F4 containing phlomispentaol A. **(B)** Fractions E5–E6 containing phlomispurtetraolone.

These individual 14 refractions E3–F4 were analyzed by HPLC-HRESIMS to obtain data on the exact mass of the compounds present in those bioactive samples. These analyses showed, in negative ionization mode, the following associated m/z ions: fractions E4 to F4 contained the molecule with m/z ions 521.3484 Da/511.3197 Da (**Figure 3**). The molecule with m/z ions 519.3328 Da/509.3040 Da was only present in the E5 and E6 fractions (**Figure 3**). The masses of the detected m/z ions for each compound differed from each other in both cases by 10,029 Da, suggesting that both compounds might be structurally related.

**Figure 4:**
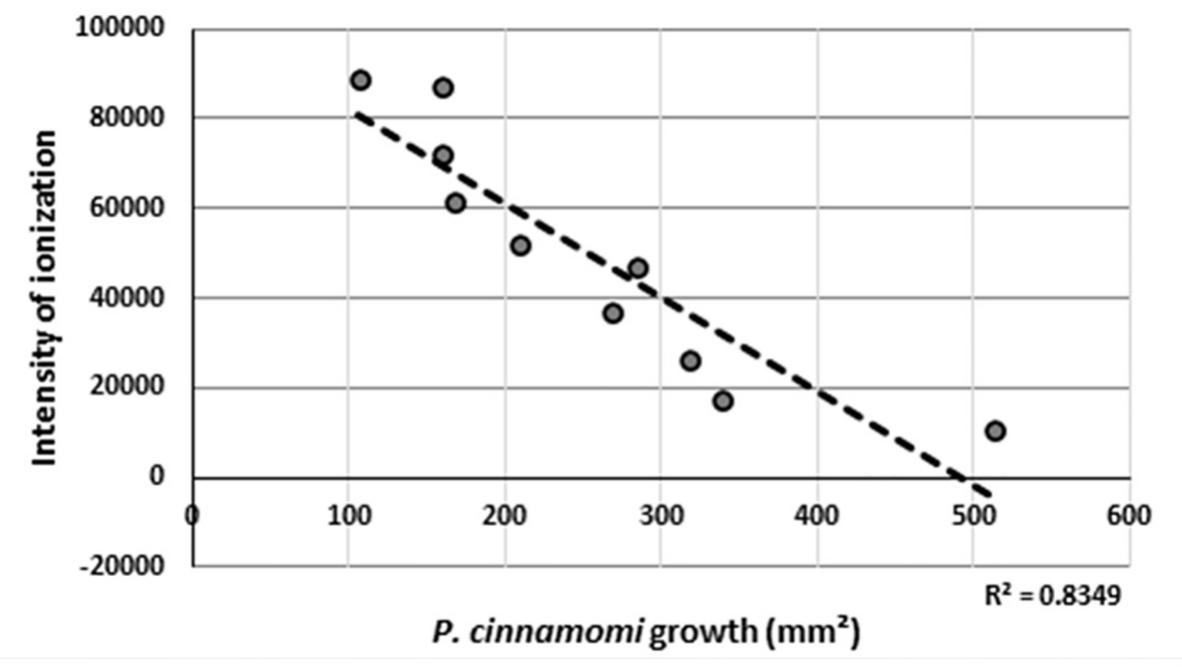
Regression line obtained between the values of ionization intensity of the detected m/z ion (521.3484 Da) in the HPLC-MS experiments and the growth of *Phytophthora cinnamomi* as measured in the bioassays.

Using the *P. cinnamomi* mycelial growth measurements obtained from the bioassay experiments, a regression graph was made (*R* ^2 = ^0.8349, *p*-value = 0.000217998), and this showed that the concentration of molecule with m/z ions of 521.3484 Da matched perfectly to the growth observed in the bioassay from each E4 to F4 fraction (**Figure 4**). Fractions E7–F4 were pooled in one vial. The solvent was removed by evaporating it under vacuum, and the remaining solid was weighed, obtaining 7.5 mg. In another vial, fractions E5 and E6 were combined, and after removing the solvent, 0.7 mg of the sample was obtained. These two final dry samples were subjected to NMR analysis to elucidate the molecular structure of the compounds present in them.

In the MIC assay, a complete inhibition was observed in the well corresponding to 75 μg/ml of phlomispentaol A sample, which corresponds to a concentration of 157.33 μM, which is therefore the MIC for this compound against *P. cinnamomi*.

### 3.2 NMR analyses

NMR analyses on the E7–F4 samples (compound A) indicated the presence of signals in the aliphatic zone of the spectrum (0.5 ppm–2 ppm in ^1^H): five singlets corresponding to methyl groups attached to a quaternary aliphatic carbon, two CH signals, and nine signals corresponding to CH_2_ (**Supplementary Figure S1**). In the 3 ppm–4.5 ppm zone, which corresponds to signals of CH*_n_* groups bound to oxygen, three CH–O signals and two CH_2_–O groups were found. A CH signal attached to a CH=C double bond was also identified at 5.78 ppm (**Supplementary Figure S1**). This analysis was based on the study of the ^1^H and edited HSQC spectra, which indicate the multiplicity of proton-bound carbons (**Table 1**).

**Table 1:** Summary of MNR data obtained for compound A.

Position	Multiplicity^a^	δH, ppm (multiplicity, *J* Hz) ^b^	δC^a^ ppm	TOCSY^c^	HMBC^d^	NOE^e^
1	CH_2_	1.7, 1.3	42.7	1, 2, 3		
2	CH(−O)	3.9 (ddd, *J* = 3.0, 4.2, 12,6)	67.0	1, 2, 3		25
3	CH(−O)	4.0 (d, *J* = 3.0)	73.6	1, 2, 3	C2, C4, C5, C1	2, 24, 23
4	C	–	48.0	–	–	
5	CH	1.6	44.9	5, 6, 7		
6	CH_2_	1.49, 1.44	19.3	5, 6, 7		
7	CH_2_	1.6, 1.4	35.1	5, 6, 7		
8	C	–	40.8	–	–	
9	CH	1.67	48.6	9, 11, 12, 18		27
10	C	–	39.0	–	–	
11	CH_2_	2.0	24.0	9, 11, 12, 18		
12	CH(=C)	5.8 (m)	118.9	9, 11, 12, 18		11
13	C=	–	143.5	–	–	
14	C	–	45.4	–	–	
15	CH_2_	1.76, 1.04	28.0	15, 16		22, 26
16	CH_2_	1.6	36.9	15, 16		29
17	C	–	51.2	–	–	
18	CH(−O)	3.9 (d)	76.2	9, 11, 12, 18	C17, C22, C13	27, 16, 19
19	CH_2_	1.96, 1.13	52.9	–	C18, C17, C20, C16, C28/C29	18, 29
20	C	–	39.9	–	–	
21	CH_2_	1.5, 1.4	42.9	21, 22		
22	CH_2_	1.6, 1.3	29.4	21, 22		15
23	CH_2_(−O)	3.7 (d, *J* = 11.4), 3.6 (d, *J* = 11.4)	64.3	23, 24	C3, C24, C4, C5	25, 6
24	CH_2_(−O)	3.9 (d, *J* = 11.1), 3.7 (d, *J* = 11.1)	68.3	23, 24	C3, C23, C4, C5	6, 5
25	CH_3_	1.0	17.4	–	C5, C9	1, 2, 12, 23, 26
26	CH_3_	0.96	17.8		C5, C7, C8, C9	6, 11, 15, 25
27	CH_3_	1.13	23.4	–	C5, C13	18, 9
28	CH_3_	1.03	30.1	–	C19, C20, C21	18, 21
29	CH_3_	1.01	30.2	–	C19, C20, C21	16, 21, 22

a Determined from HSQC, HMBC, and ^13^C spectra.

b Some relevant coupling constants are indicated.

c Spin systems determined from COSY, TOCSY, or HSQC-TOCSY analyses.

d Correlations between the protons at the corresponding positions and the indicated carbons.

e Correlations between the protons at the corresponding positions and the indicated protons.

The nature of these signals indicated that the structure of compound A was triterpenic, of the cholesteryl or phytosterol type. No peptide-type components or aromatic compounds were observed, nor did it show signs of carbohydrates.

**Figure 5:**
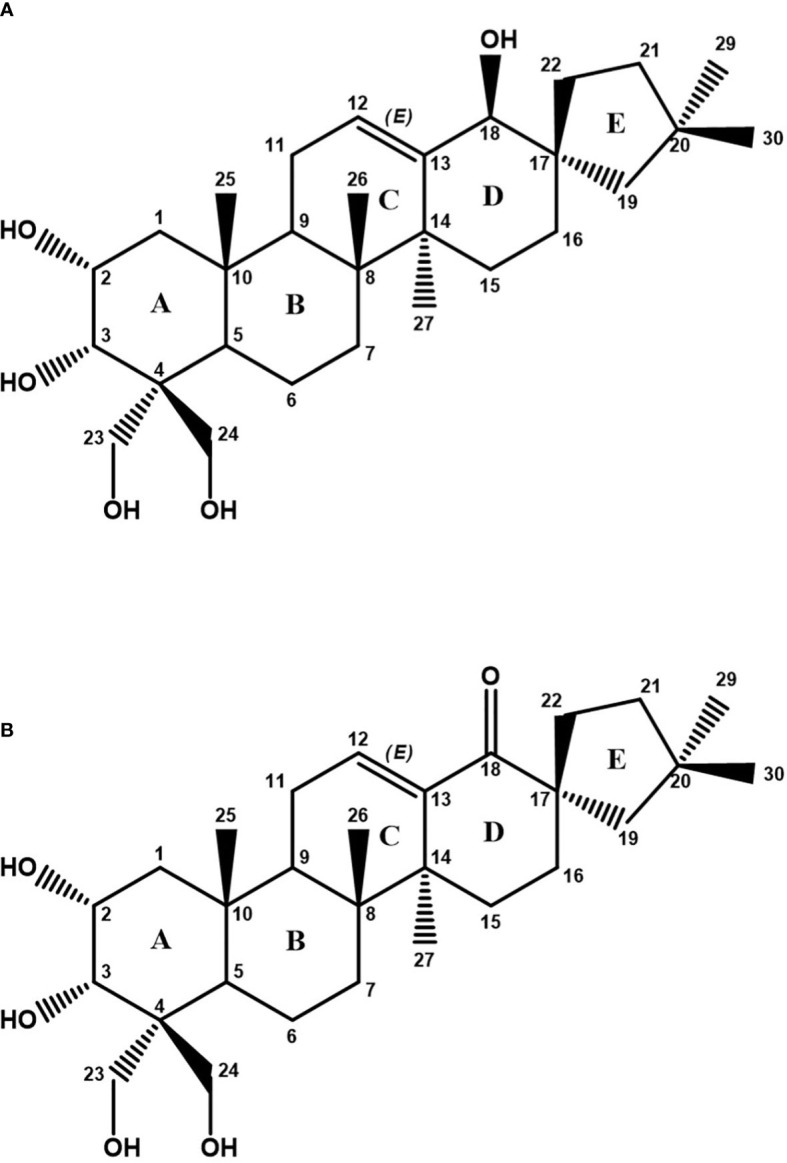
**(A)** Chemical structure of phlomispentaol A (compound A). **(B)** Chemical structure of phlomispurtetraolone (compound B) as deduced from NMR analyses.

The detailed analysis of the ^13^C, HSQC, and HMBC spectra allowed the identification of six aliphatic quaternary carbons and one quaternary carbon of olefinic type. The different spin systems were identified by experiments of scalar correlation, COSY, TOCSY, and HSQC-TOCSY. These could be ordered and related to each other through the information obtained from long-distance ^1^H-^13^C correlation (HMBC) experiments and through experiments of dipolar correlation based on the Nuclear Overhauser Effect (NOESY or ROESY). The information provided in this last type of dipole correlation experiment, together with the study of the vicinal coupling constants ^1^H-^1^H (at three bonds), allowed the assignment of the stereochemistry of the different stereogenic centers of the compound A molecule (**Table 1**; **Supplementary Figure S1**). All this information allowed us to verify that compound A was a spirocyclic nortriterpenoid (**Figure 5A**). Its exact mass is 476.3502 Da, and its molecular formula is C_29_H_48_O_5_. Based on this information, it was possible to explain the masses found in the HPLC-MS experiments: the m/z ion 521.3484 Da is an adduct of the type [M+COOH]−, and the m/z ion 511.3197 Da is an adduct of the type [M+Cl]−. Both adducts were formed from compound A during the ionization process in the HPLC-MS equipment. The structure of this compound A corresponds to phlomispentaol A, previously described as an antitumor compound in extracts from a plant from the same taxonomic genus, *Phlomis umbrosa* (Liu et al., 2008).

NMR analyses on the E5–E6 samples (compound B) showed a ^1^H NMR spectrum in which phlomispentaol A was also identified. In addition, a new compound B of a similar nature appeared in an approximate ratio of 1:2 (compound B: phlomispentaol A). The small amount of sample (0.7 mg) and the minor proportion of compound B in the mixture meant that it was not possible to identify and assign all the signals of this compound, but it was possible to determine some of the differences existing between both samples. In the COSY, TOCSY, and HSQC-TOCSY scalar correlation experiments, for compound B, it was observed that there was no correlation between the olefinic proton and any other proton in the CH*_n_*–O group zone. In the HMBC experiment, a new quaternary ^13^C signal appeared at 208.3 ppm, corresponding to a ketone group. The new signals corresponding to the CH_2_ group at carbon 19 (in the structure of compound B) were those that correlate with the ketone carbon (**Supplementary Figure S2**). The analysis of ^1^H-^13^C-HSQC spectra from compound B allowed us to make several comparisons with the signals from phlomispentaol A spectra (**Supplementary Figure S3**). Following these analyses, a structural formula was proposed for compound B (phlomispurtetraolone, **Figure 5B**). It is a spirocyclic nortriterpenoid identical to phlomispentaol A, except for having the C–OH group of carbon 18 oxidized to a ketone group (C=O), and this novel triterpenoid was therefore named phlomispurtetraolone. Its exact mass is 474.3345 Da, and its molecular formula is C_29_H_46_O_5_. This information made it possible to explain the origin of the m/z ions of 519.3328 Da/509.3040 Da found in the HPLC-MS experiments: the m/z ion of 519.3328 Da is a [M+COOH]−-type adduct derived from compound B, and the m/z ion of 509.3040 Da is a [M+Cl]−-type adduct as well of compound B.

## 4 Discussion

**Figure 6:**
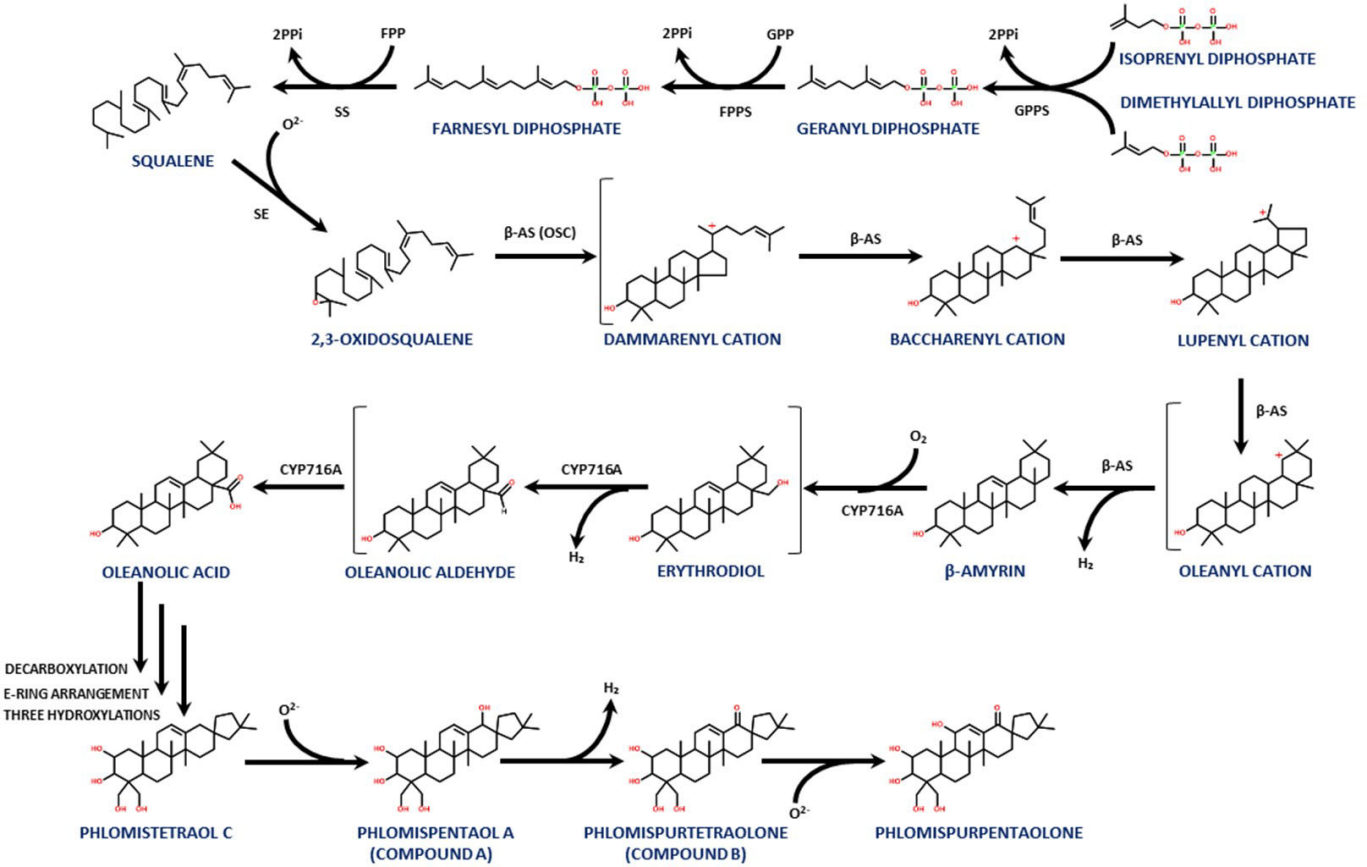
A proposed biosynthetic pathway for this family of triterpenoids, showing the steps from IPP and DMAPP to phlomispentaol A and phlomispurtetraolone, as well as its putative derivative, phlomispurpentaolone.

The biosynthetic pathway that leads to terpenoid molecules, like the phlomispentaol A and phlomispurtetraolone described above, has several common steps that start when the geranyl pyrophosphate synthase (GPPS) binds dimethylallyl diphosphate (DMAPP) to isoprenyl diphosphate (IPP), generating geranyl diphosphate (GPP) (**Figure 6**). These two universal biosynthetic precursors for terpenoids (DMAPP and IPP) are generated from mevalonate in plants. GPP is bound to another molecule of IPP by the farnesyl pyrophosphate synthase (FPPS), generating farnesyl pyrophosphate (FPP). The squalene synthase (SS) then binds two molecules of FPP, generating squalene (C30). The squalene epoxidase (SE) converts squalene into 2,3-oxidosqualene, which is the common precursor of several triterpenoids in plants (Gosh, 2016; Yang et al., 2017) (**Figure 6**).

The carbon skeleton of the triterpenoid produced by any given plant will depend on the type of 2,3-oxidosqualene cyclase (OSC) present in its tissues. In this case, the β-amyrin synthase (a type of OSC) transforms the 2,3-oxidosqualene into β-amyrin, through the intermediates dammarenyl, baccharenyl, lupenyl, and oleanyl cations (Kushiro et al., 2000; Xue et al., 2012; Babalola and Shode, 2013; Prasad et al., 2019) (**Figure 6**). This β-amyrin intermediate is active against *Candida albicans*, exhibiting antifungal cytotoxic activity through an increase in intracellular reactive oxygen species and calcium homeostasis disturbance in this pathogenic yeast (Johann et al., 2007; Kwun et al., 2021).

From β-amyrin, two consecutive oxidations at C28 performed by a cytochrome P_450_ monooxygenase (CYP) from the CYP716A subfamily convert β-amyrin into oleanolic acid. The transformation of β-amyrin into oleanolic acid involves the intermediates erythrodiol and oleanolic aldehyde (Fukushima et al., 2011; Huang et al., 2012; Jo et al., 2017; Castellano et al., 2022). Interestingly, oleanolic acid can be extracted from other species of this genus, like *Phlomis cashmeriana*, as reported by Hussain et al. (2010). This molecule showed antimicrobial activity against several plant pathogens, such as *Phytophthora nicotianae*, *Penicillium expansum*, *Aspergillus parasiticus*, and *Colletotrichum gloeosporioides*, with a MIC value between 7.8 and 15.6 μg/mL (Eloff et al., 2017). These pathogenic species affect apples, avocados, citrus fruits, corn, and other plants. Oleanolic acid was also found to be active against *Penicillium ochrochloron*, *Penicillium foniculosum*, *Aspergillus ochraceus*, *Aspergillus flavus*, *Aspergillus niger*, *Aspergillus versicolor*, and *Candida albicans* (Yessoufou et al., 2015), including three *C. albicans* strains that are resistant against common treatment drugs such as fluconazole (Yessoufou et al., 2015; Eloff et al., 2017; Harley et al., 2021). The antifungal activity of oleanolic acid (as well as its close relative, ursolic acid) has been associated with inhibition of cell wall biosynthesis components, indicating that the presence of the COOH group is important for this bioactivity (Schwartz, 2001).

Also, in *Phlomis umbrosa*, phlomispentaol A was described together with its structurally related phlomistetraol C, which lacks the hydroxyl group at C18. Phlomispentaol A was described as an antitumor, especially against HeLa (cervical epithelium) and L929 (fibroblasts) cancer cell lines, and as moderately cytotoxic against the HL-60 (leukemia) cancer cell line (Liu et al., 2008; Le et al., 2018). Another structurally related triterpenoid from this plant, phlomisu E, which contains an extra hydroxyl group at C19 and an aldehyde at C23 (in comparison with phlomispentaol A), showed high cytotoxic activity against HeLa, HL-60, and MCF-7 (breast cancer) cell lines (Le et al., 2018). Liu et al. (2008) proposed a biosynthetic pathway for these nortriterpenoids with oleanolic acid as the common precursor, involving an initial decarboxylation on oleanolic acid (at rings D–E) that may be followed by a rearrangement of the E ring, in which C22, initially bound to C18, now binds to C17 (transforming the initial six-membered ring E into a five-membered one). Later, four hydroxylations are needed to biosynthesize phlomispentaol A at positions C2, C23, C24, and C18 (Liu et al., 2008).

Phlomispentaol A was also found in the methanolic extracts of the plant *Phlomis stewartii*, together with three other nortriterpene bioactive compounds with the same E ring arrangement, all of them showing inhibition against α-glucosidase and therefore being potential drugs for type II diabetes treatment (Riaz et al., 2019). Also, in *P. stewartia* extracts, the nortriterpenoid stewartiisin A, which is identical to phlomispentaol A but lacking the hydroxyl group at C2, showed much better inhibitory activity against α-glucosidase (IC_50 = _38.0 μM). The intermediate oleanolic acid in these extracts was also found to be a moderate inhibitor of α-glucosidase (Jabeen et al., 2013).

In the present work, the presence of phlomispurtetraolone indicates the need for an oxidation step acting on the hydroxyl group at C18 of phlomispentaol A, introducing a keto group at this C18. Interestingly, Mateus et al. (2016), working as well with *Phlomis purpurea* root extracts, identified another nortriterpenoid, named phlomispurpentaolone, in which the only difference with phlomispurtetraolone is an extra hydroxylation at C11. This molecule, phlomispurpentaolone, also showed inhibitory effects on the pathogen *P. cinnamomi* and cytotoxic activity *in vitro* against HeLa cell lines and 929 cells (IC_50 = _22 µM and IC_50 = _50 µM, respectively) (Mateus et al., 2016). This plant has been analyzed to determine which metabolite groups suffer major changes in their levels over a challenging period of 72 h with *P. cinnamomi*. Interestingly, at the root tissue, the presence of the pathogen induces the overproduction of terpenoids, polyketides, prenol lipids, alkaloids derived from tryptophan, and flavonoids (Neves et al., 2023). Terpenoid biosynthetic genes in this plant were also upregulated at the transcriptional level after induction with this plant pathogen (Baldé et al., 2017).

Nortriterpenoids similar to phlomispentaol A, denominated norviscoside (lacking the three hydroxyl groups at C2, C23, and C24, having the hydroxyl group at C3 oxidized to a keto group, and showing the presence of an extra keto group at C19 and a glucosylated carboxyl group at C25 instead of the canonical methyl group over there), and norviscoside heptaacetate, can be found in *Phlomis viscosa* plant extracts (Çaliş et al., 2004). Knowing all this information, we propose a new step forward for the biosynthetic pathway previously described for this family of bioactive terpenoids derived from oleanolic acid (**Figure 6**).

Therefore, in this study, we have isolated and characterized two 28-noroleane spirocyclic triterpenoids from organic extracts of the roots of *P. purpurea*, with one of them, phlomispurtetraolone (compound B), being first described in this work. These two molecules are members of a biosynthetic family that contains various antimicrobial compounds and has oleanolic acid as the common precursor. One of these compounds, phlomispentaol A, was found to be active against the phytopathogen *P. cinnamomi* in this work. This information can be used to propose these molecules as a promising treatment to solve the worldwide problem caused by this plague. Further studies are needed to determine the best way to apply this treatment and detect possible side effects.

## 5 Species

*Phytophthora cinnamomi* Rands. NCBI Taxonomy ID 4785. *Phlomis purpurea* NCBI Taxonomy ID 316258.

## Data availability statement

The original contributions presented in the study are included in the article/**Supplementary Material**. Further inquiries can be directed to the corresponding author.

## Author contributions

LF-C and MG-D carried out the experimental analyses and interpretation of the data. BR, JM, CV, and FL designed the work. LF-C, CV, and FL drafted and revised the manuscript. BR, JM, and FL obtained the funding for carrying out the experimental work. All authors contributed to the article and approved the submitted version.
