# Supplementary material for: Isolation of two triterpenoids from Phlomis purpurea, one of them with anti-oomycete activity against Phytophthora cinnamomi, and insights into its biosynthetic pathway
Source: Front Plant Sci. 2023 Aug 25;14:1180808. doi: 10.3389/fpls.2023.1180808 (PMC10485375; doi:10.3389/fpls.2023.1180808)

Isolation of a triterpenoid from *Phlomis purpurea* with anti-oomycete activity against *Phytophthora cinnamomi* and insights into its biosynthetic pathway

L. Fernández-Calleja[1,2,3], M. García-Domínguez[1,2,3], B. Isabel Redondo[4], J.L. Gómez Martín[5], C.J. Villar[1,2,3], F. Lombó[1,2,3*]

[1] Research Unit "Biotechnology in Nutraceuticals and Bioactive Compounds-BIONUC", Departamento de Biología Funcional, Área de Microbiología, Universidad de Oviedo, Oviedo, Spain

[2] Instituto Universitario de Oncología del Principado de Asturias, Oviedo, Spain

[3] Instituto de Investigación Sanitaria del Principado de Asturias, Oviedo, Spain

[4] Department Animal Science, Faculty of Veterinary Medicine, Universidad Complutense de Madrid, Madrid, Spain

[5]Research and Development Department, Campojerez SL, Jerez de los Caballeros, Badajoz, Spain.

*** Correspondence:**
Corresponding Author
lombofelipe@uniovi.es

Species: *Phytophthora cinnamomi* Rands. NCBI Taxonomy ID 4785. *Phlomis purpurea* NCBI Taxonomy ID 316258.

Keywords: anti-oomycete, root rot, natural compound, oomycete, dieback.

Supplementary Material Figures

Figure S1: NMR spectra of E7-F4 sample (phlomispentaol A). S1A to S1F: ^{1}H, S1G: TOCSY, S1H: HSQC, S1I to S1M: ^{13}C.

S1A

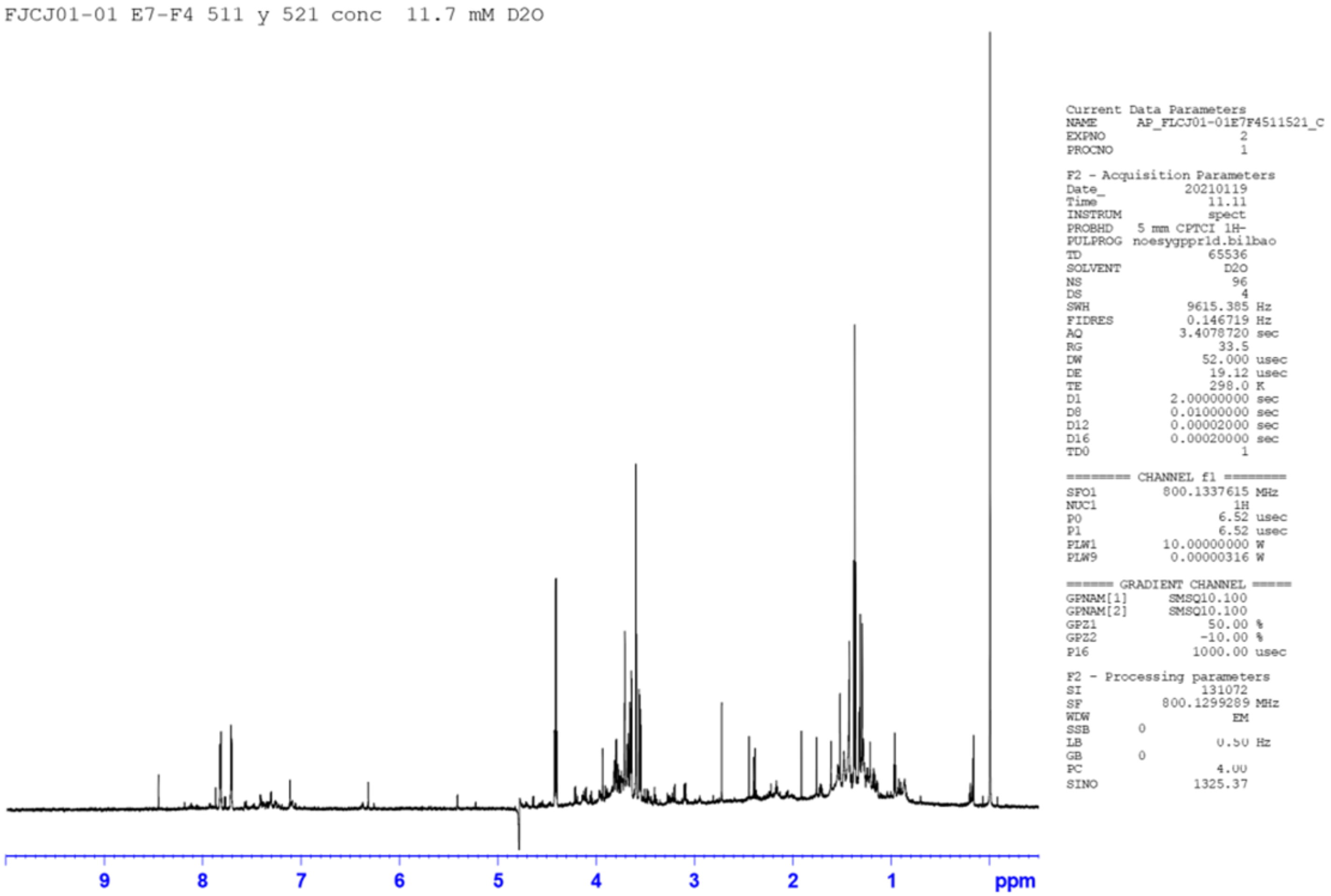

S1B

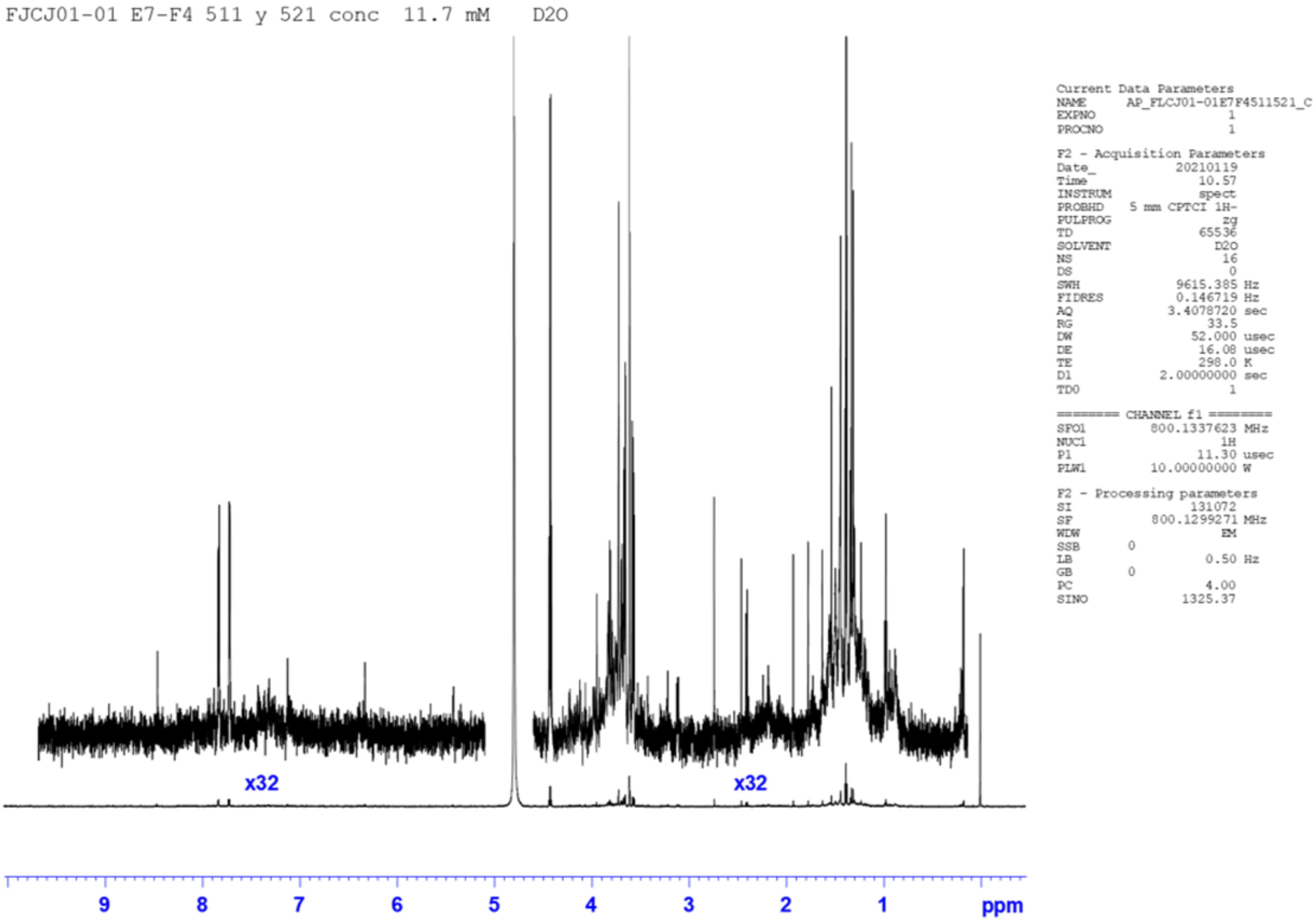
FJCJ01-01 E7-F4 511 y 521 conc 11.7 mM D2O
x32
x32
9
8
7
6
5
4
3
2
1
ppm
Current Data Parameters
NAME AP_FLCJ01-01E7F4511521_C
EXPNO 1
PROCNO 1
F2 - Acquisition Parameters
Date_ 20210119
Time 10.57
INSTRUM spect
PROBHD 5 mm CPTCI 1H-
PULPROG zg
TD 65536
SOLVENT D2O
NS 16
DS 0
SWH 9615.385 Hz
FIDRES 0.146719 Hz
AQ 3.4078720 sec
RG 33.5
DW 52.000 usec
DE 16.08 usec
TE 298.0 K
D1 2.00000000 sec
TD0 1
======== CHANNEL f1 ========
SFO1 800.1337623 MHz
NUC1 1H
P1 11.30 usec
PLW1 10.00000000 W
F2 - Processing parameters
SI 131072
SF 800.1299271 MHz
WDW EM
SSB 0
LB 0.50 Hz
GB 0
PC 4.00
SINO 1325.37

S1C

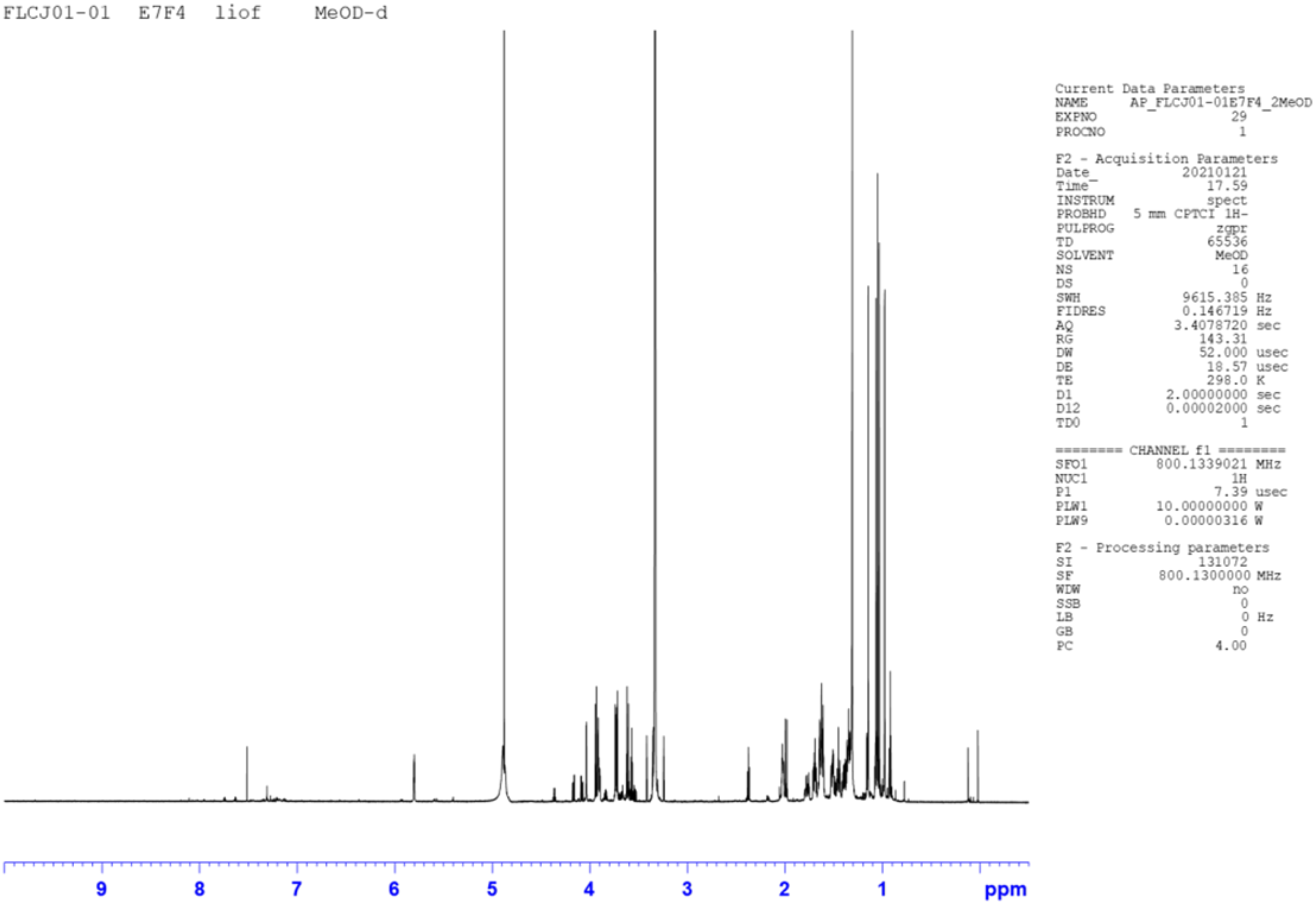
FLCJ01-01 E7F4 liof MeOD-d
Current Data Parameters
NAME AP_FLCJ01-01E7F4_2MeOD
EXPNO 29
PROCNO 1
F2 - Acquisition Parameters
Date_ 20210121
Time_ 17.59
INSTRUM spect
PROBHD 5 mm CPTCI 1H-
PULPROG zgpr
TD 65536
SOLVENT MeOD
NS 16
DS 0
SWH 9615.385 Hz
FIDRES 0.146719 Hz
AQ 3.4078720 sec
RG 143.31
DW 52.000 usec
DE 18.57 usec
TE 298.0 K
D1 2.00000000 sec
D12 0.00002000 sec
TD0 1
======== CHANNEL f1 ========
SFO1 800.1339021 MHz
NUC1 1H
P1 7.39 usec
PLW1 10.00000000 W
PLW9 0.00000316 W
F2 - Processing parameters
SI 131072
SF 800.1300000 MHz
WDW no
SSB 0
LB 0 Hz
GB 0
PC 4.00
9
8
7
6
5
4
3
2
1
ppm

S1D

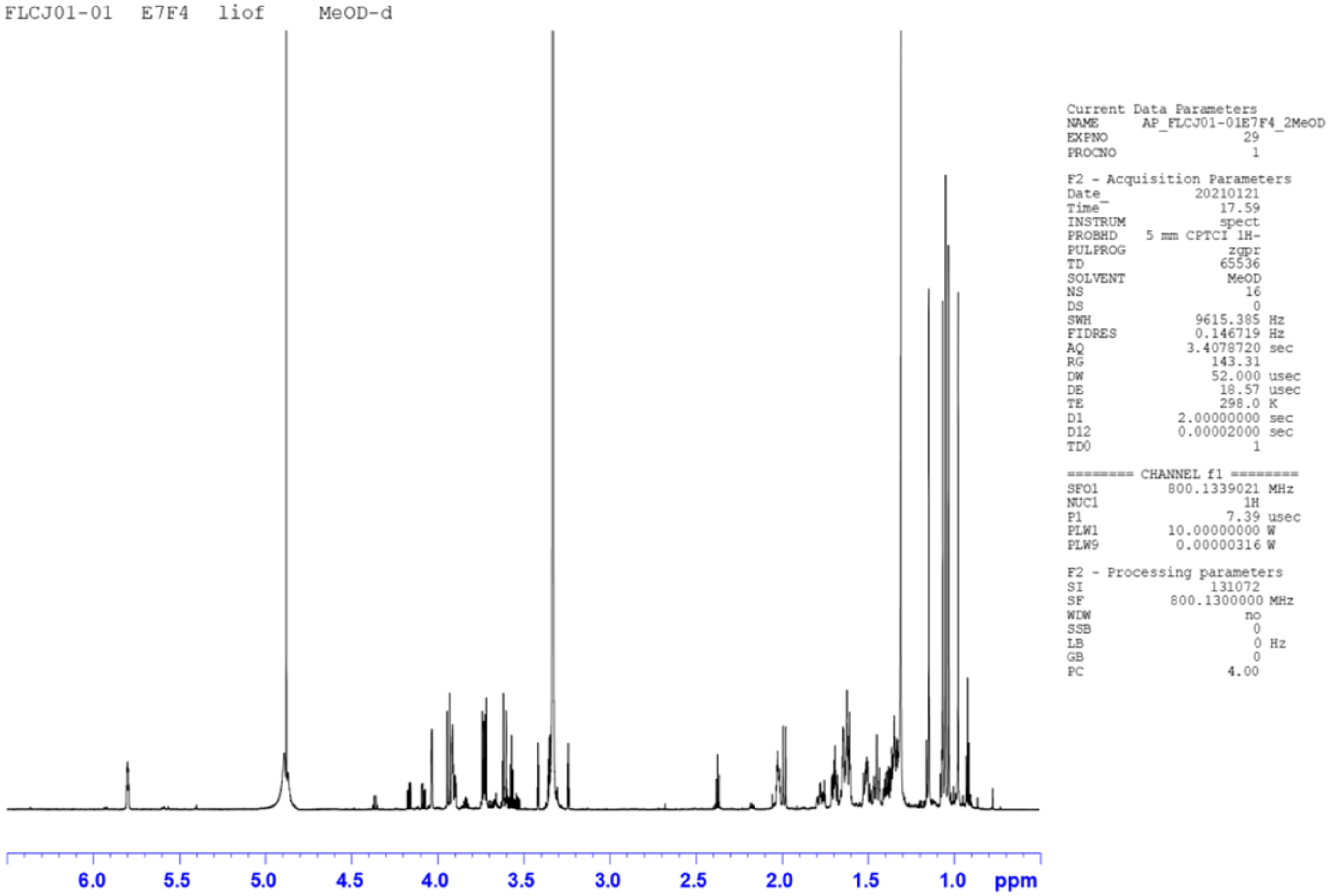
FLCJ01-01 E7F4 liof MeOD-d
Current Data Parameters
NAME AP_FLCJ01-01E7F4_2MeOD
EXPNO 29
PROCNO 1
F2 - Acquisition Parameters
Date_ 20210121
Time 17.59
INSTRUM spect
PROBHD 5 mm CPTCI 1H-
PULPROG zgpr
TD 65536
SOLVENT MeOD
NS 16
DS 0
SWH 9615.385 Hz
FIDRES 0.146719 Hz
AQ 3.4078720 sec
RG 143.31
DW 52.000 usec
DE 18.57 usec
TE 298.0 K
D1 2.00000000 sec
D12 0.00002000 sec
TD0 1
======== CHANNEL f1 ========
SFO1 800.1339021 MHz
NUC1 1H
P1 7.39 usec
PLW1 10.00000000 W
PLW9 0.00000316 W
F2 - Processing parameters
SI 131072
SF 800.1300000 MHz
WDW no
SSB 0
LB 0 Hz
GB 0
PC 4.00
6.0
5.5
5.0
4.5
4.0
3.5
3.0
2.5
2.0
1.5
1.0
ppm

S1E

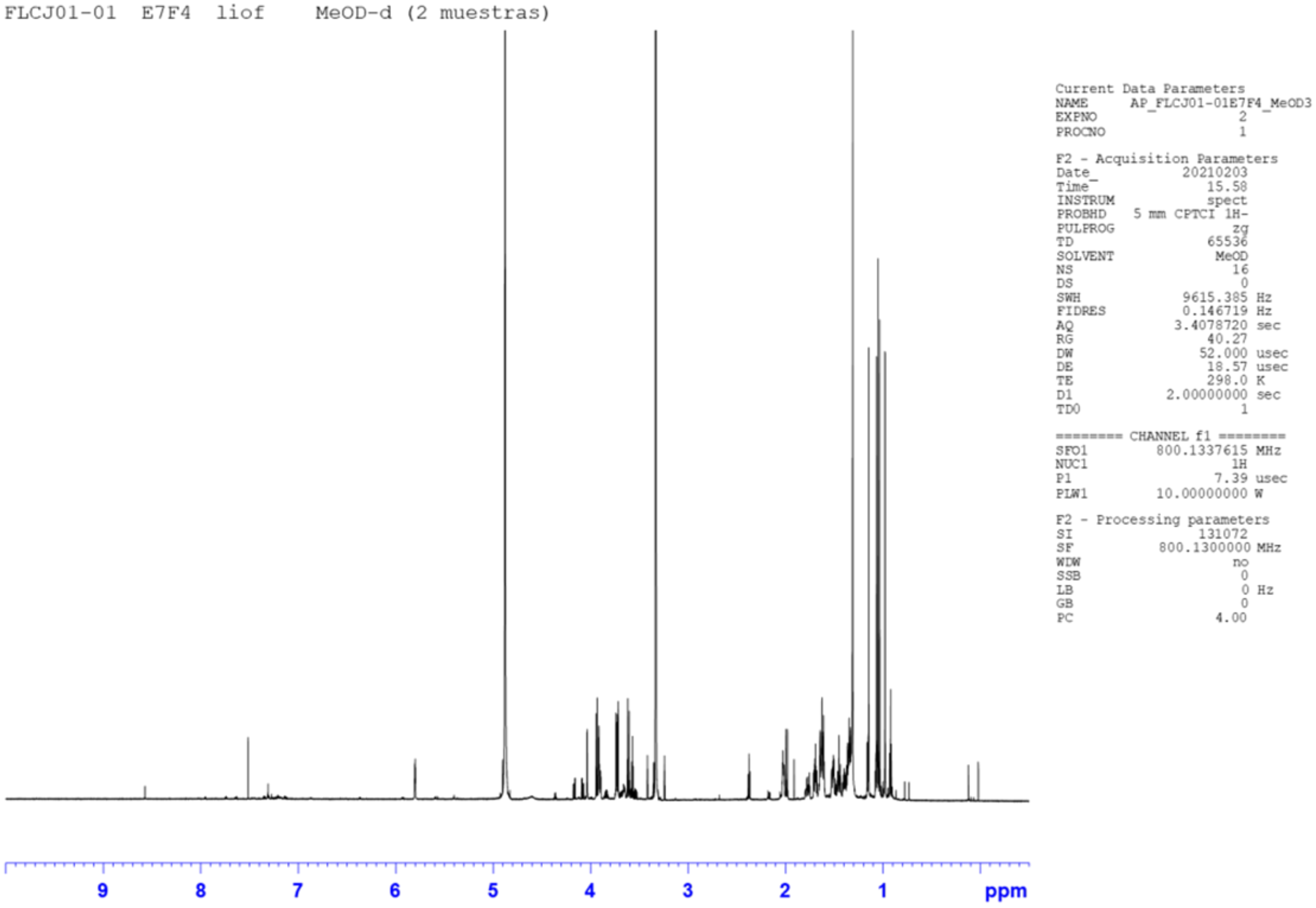
FLCJ01-01 E7F4 liof MeOD-d (2 muestras)
Current Data Parameters
NAME AP_FLCJ01-01E7F4_MeOD3
EXPNO 2
PROCNO 1
F2 - Acquisition Parameters
Date_ 20210203
Time_ 15.58
INSTRUM spect
PROBHD 5 mm CPTCI 1H-
PULPROG zg
TD 65536
SOLVENT MeOD
NS 16
DS 0
SWH 9615.385 Hz
FIDRES 0.146719 Hz
AQ 3.4078720 sec
RG 40.27
DW 52.000 usec
DE 18.57 usec
TE 298.0 K
D1 2.00000000 sec
TD0 1
======== CHANNEL f1 ========
SFO1 800.1337615 MHz
NUC1 1H
P1 7.39 usec
PLW1 10.00000000 W
F2 - Processing parameters
SI 131072
SF 800.1300000 MHz
WDW no
SSB 0
LB 0 Hz
GB 0
PC 4.00
9
8
7
6
5
4
3
2
1
ppm

S1F

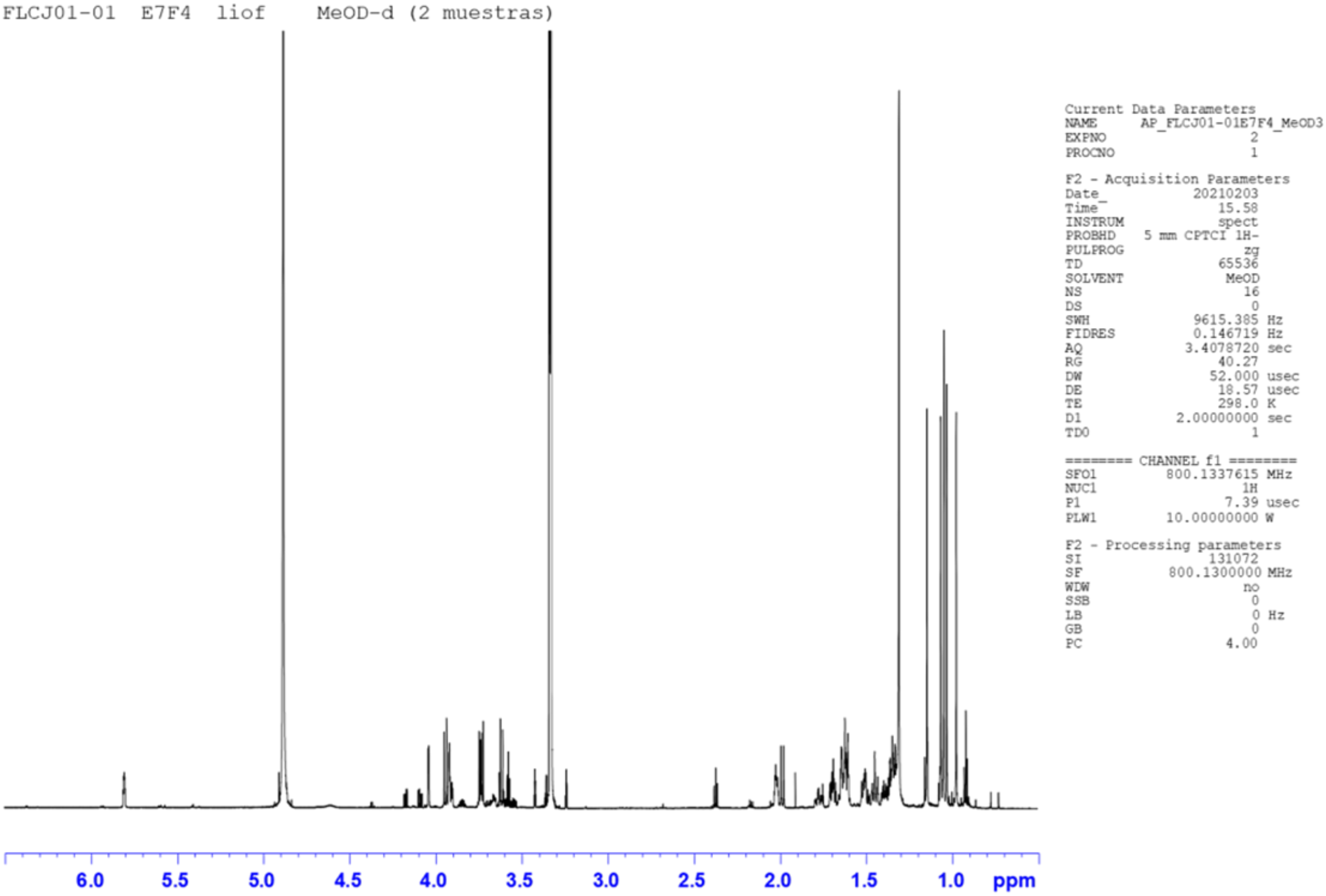
FLCJ01-01 E7F4 liof MeOD-d (2 muestras)
Current Data Parameters
NAME AP_FLCJ01-01E7F4_MeOD3
EXPNO 2
PROCNO 1
F2 - Acquisition Parameters
Date_ 20210203
Time 15.58
INSTRUM spect
PROBHD 5 mm CPTCI 1H-
PULPROG zg
TD 65536
SOLVENT MeOD
NS 16
DS 0
SWH 9615.385 Hz
FIDRES 0.146719 Hz
AQ 3.4078720 sec
RG 40.27
DW 52.000 usec
DE 18.57 usec
TE 298.0 K
D1 2.00000000 sec
TD0 1
======== CHANNEL f1 ========
SFO1 800.1337615 MHz
NUC1 1H
P1 7.39 usec
PLW1 10.00000000 W
F2 - Processing parameters
SI 131072
SF 800.1300000 MHz
WDW no
SSB 0
LB 0 Hz
GB 0
PC 4.00
6.0
5.5
5.0
4.5
4.0
3.5
3.0
2.5
2.0
1.5
1.0
ppm

SIG

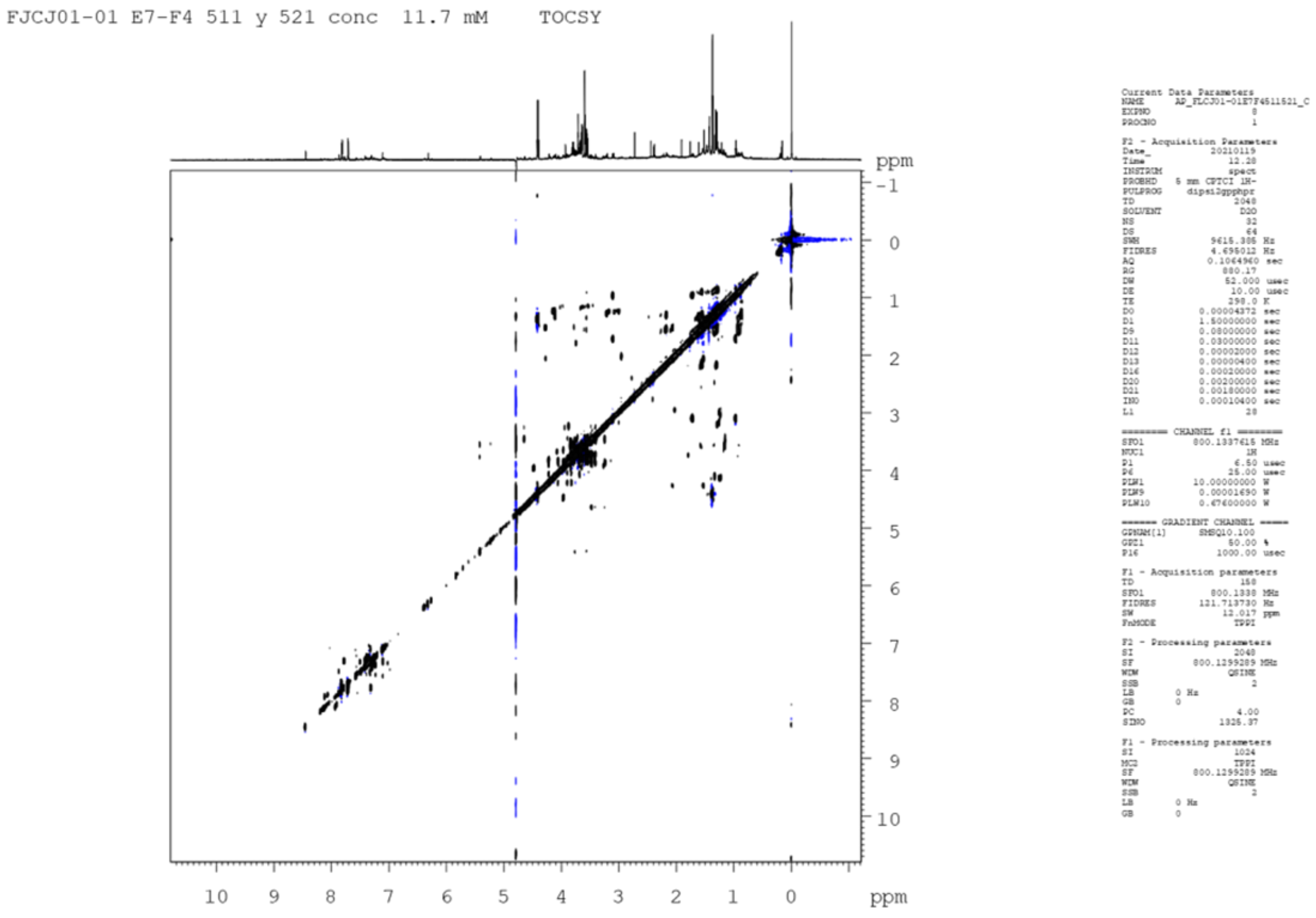

FJCJ01-01 E7-F4 511 y 521 conc 11.7 mM TOCSY
ppm
-1
0
1
2
3
4
5
6
7
8
9
10
10 9 8 7 6 5 4 3 2 1 0 ppm
Current Data Parameters
NAME AP_FLCJ01-01E7F4511521_C
EXPNO 8
PROCNO 1
F2 - Acquisition Parameters
Date_ 20210119
Time 12.28
INSTRUM spect
PROBHD 5 mm CPTCI 1H-
PULPROG dipsi2gpphpr
TD 2048
SOLVENT D2O
NS 32
DS 64
SWH 9615.385 Hz
FIDRES 4.695012 Hz
AQ 0.1064960 sec
RG 880.17
DW 52.000 usec
DE 10.00 usec
TE 298.0 K
D0 0.00004372 sec
D1 1.50000000 sec
D9 0.08000000 sec
D11 0.03000000 sec
D12 0.00002000 sec
D13 0.00000400 sec
D16 0.00020000 sec
D20 0.00200000 sec
D21 0.00180000 sec
IN0 0.00010400 sec
L1 28
======== CHANNEL f1 ========
SFO1 800.1337615 MHz
NUC1 1H
P1 6.50 usec
P6 25.00 usec
PLW1 10.00000000 W
PLW9 0.00001690 W
PLW10 0.47600000 W
====== GRADIENT CHANNEL =====
GPNAM[1] SMSQ10.100
GPZ1 50.00 %
P16 1000.00 usec
F1 - Acquisition parameters
TD 158
SFO1 800.1338 MHz
FIDRES 121.713730 Hz
SW 12.017 ppm
FnMODE TPPI
F2 - Processing parameters
SI 2048
SF 800.1299289 MHz
WDW QSINE
SSB 2
LB 0 Hz
GB 0
PC 4.00
SINO 1325.37
F1 - Processing parameters
SI 1024
MC2 TPPI
SF 800.1299289 MHz
WDW QSINE
SSB 2
LB 0 Hz
GB 0

S1H

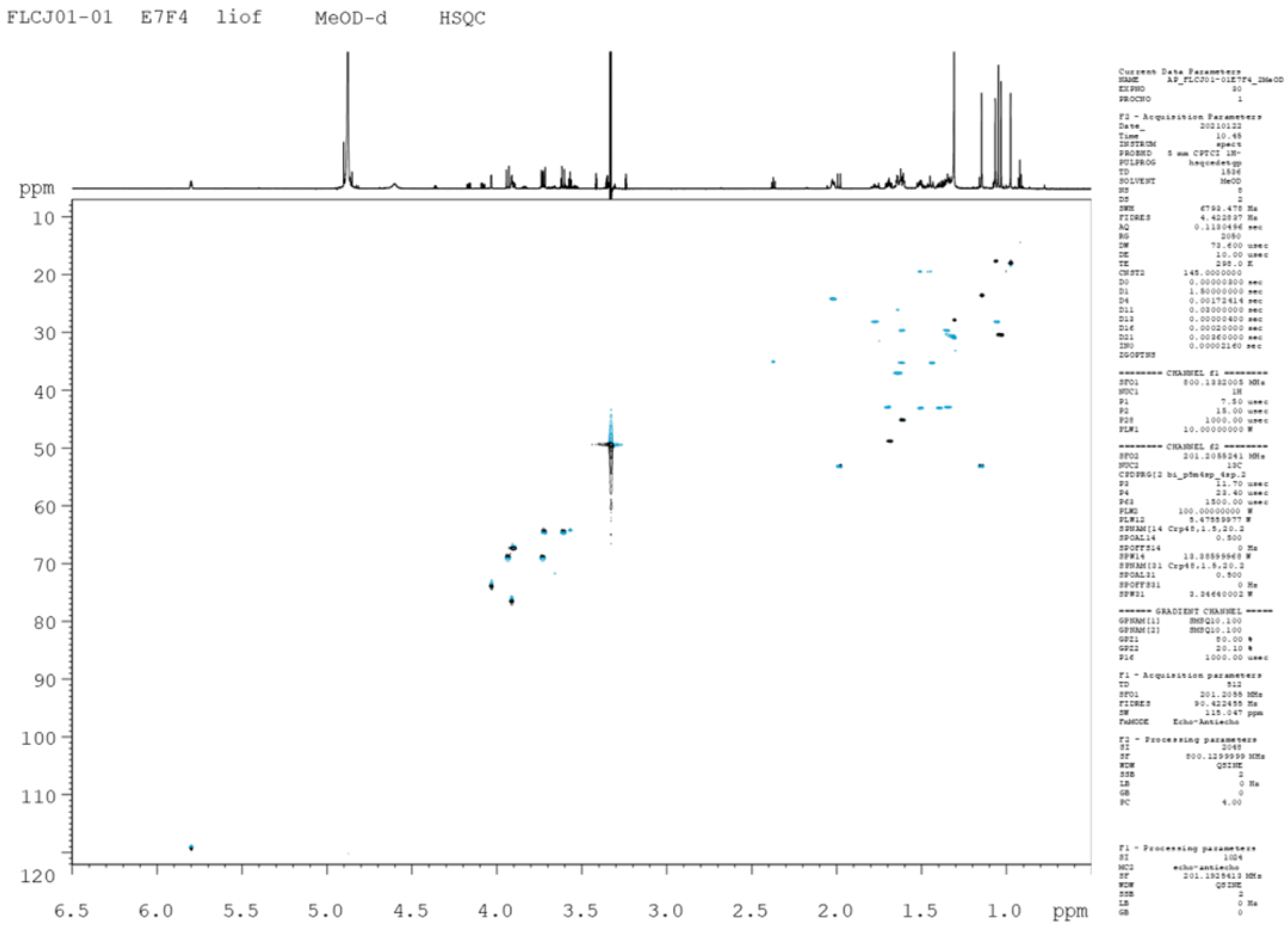

S1I

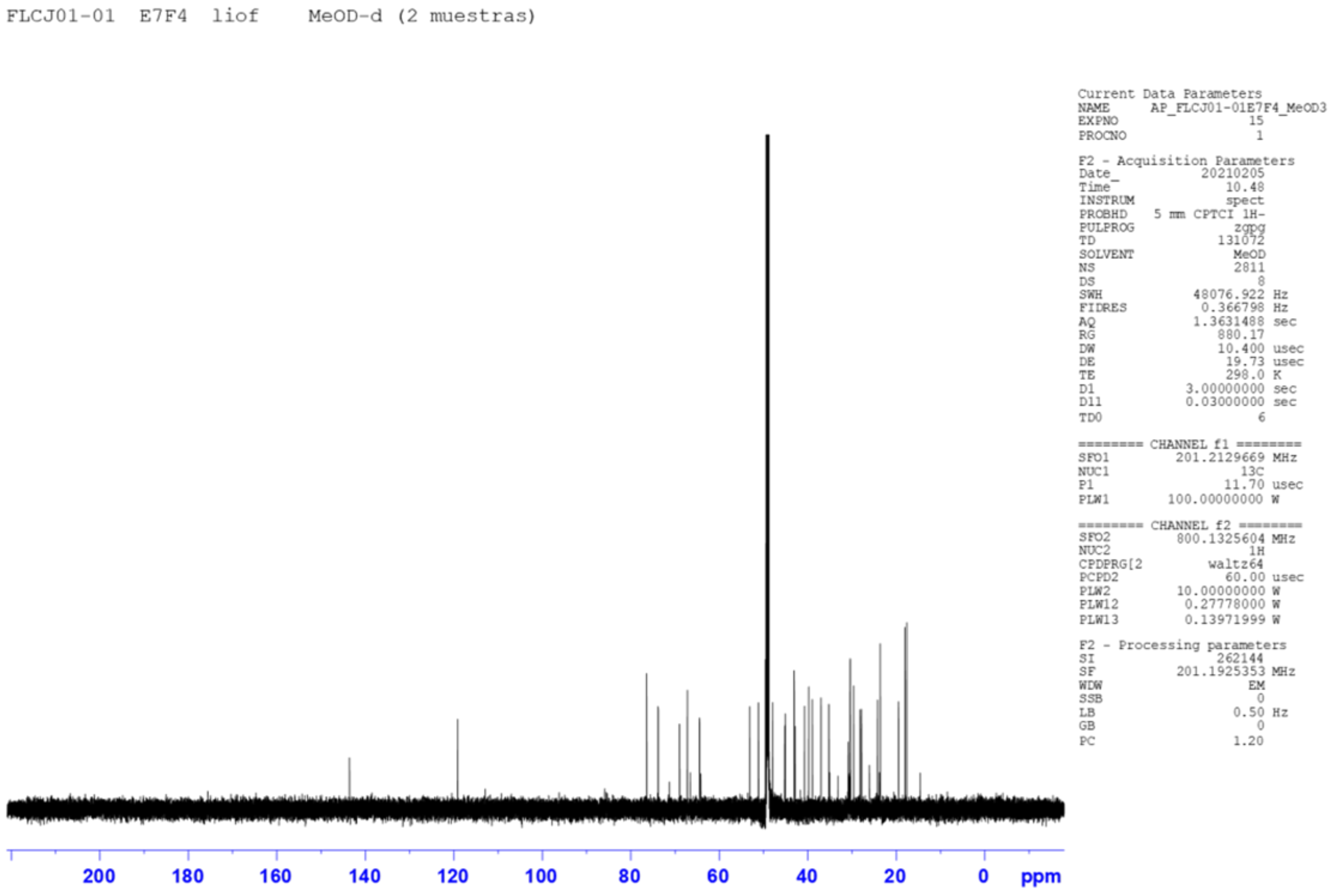
FLCJ01-01 E7F4 liof MeOD-d (2 muestras)
Current Data Parameters
NAME AP_FLCJ01-01E7F4_MeOD3
EXPNO 15
PROCNO 1
F2 - Acquisition Parameters
Date_ 20210205
Time 10.48
INSTRUM spect
PROBHD 5 mm CPTCI 1H-
PULPROG zgpg
TD 131072
SOLVENT MeOD
NS 2811
DS 8
SWH 48076.922 Hz
FIDRES 0.366798 Hz
AQ 1.3631488 sec
RG 880.17
DW 10.400 usec
DE 19.73 usec
TE 298.0 K
D1 3.00000000 sec
D11 0.03000000 sec
TD0 6
======== CHANNEL f1 ========
SFO1 201.2129669 MHz
NUC1 13C
P1 11.70 usec
PLW1 100.00000000 W
======== CHANNEL f2 ========
SFO2 800.1325604 MHz
NUC2 1H
CPDPRG[2 waltz64
PCPD2 60.00 usec
PLW2 10.00000000 W
PLW12 0.27778000 W
PLW13 0.13971999 W
F2 - Processing parameters
SI 262144
SF 201.1925353 MHz
WDW EM
SSB 0
LB 0.50 Hz
GB 0
PC 1.20
200
180
160
140
120
100
80
60
40
20
0
ppm

SIJ

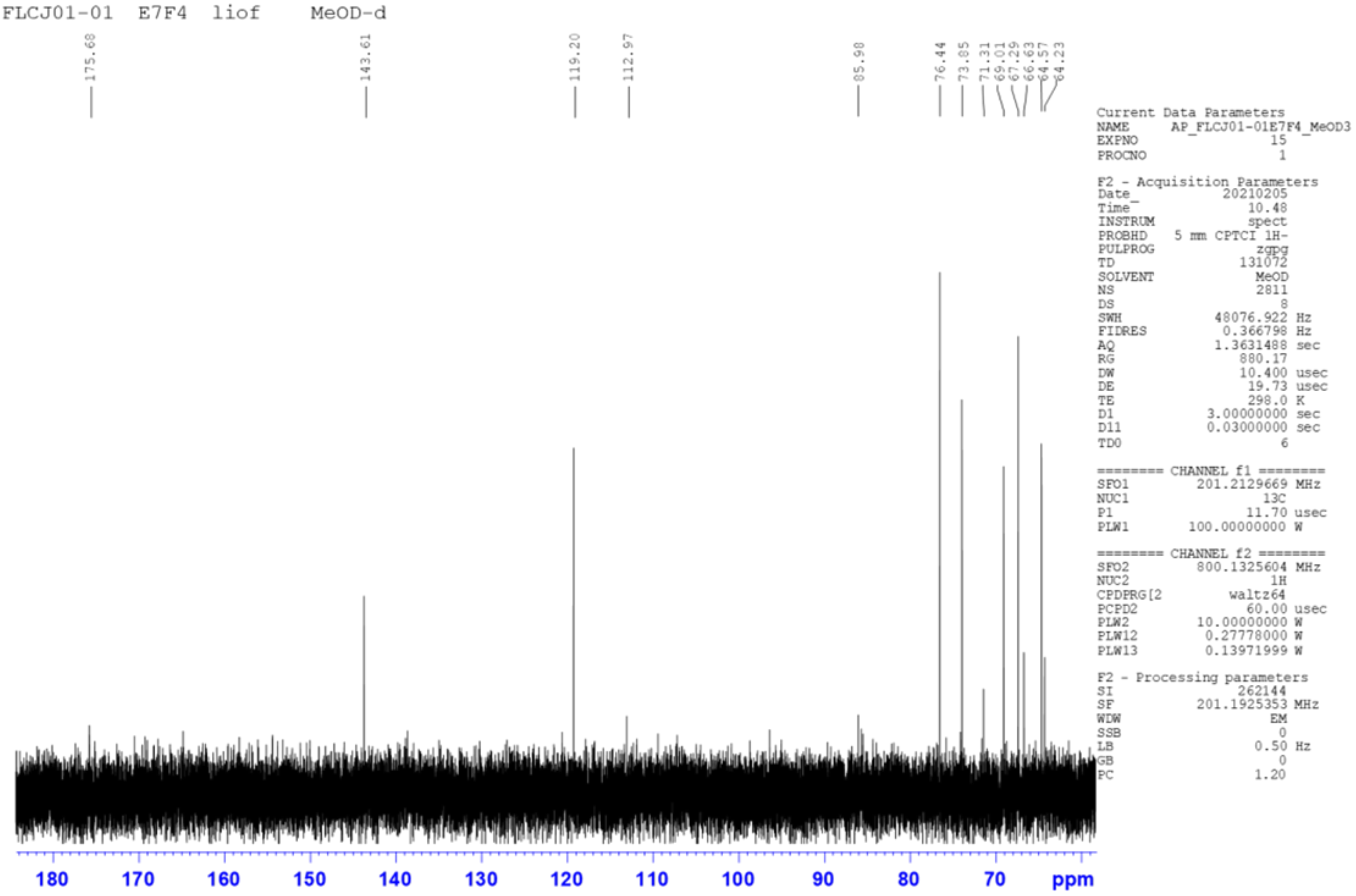
FLCJ01-01 E7F4 liof MeOD-d
175.68
143.61
119.20
112.97
85.98
76.44
73.85
71.31
69.01
67.29
66.63
64.57
64.23
Current Data Parameters
NAME AP_FLCJ01-01E7F4_MeOD3
EXPNO 15
PROCNO 1
F2 - Acquisition Parameters
Date_ 20210205
Time 10.48
INSTRUM spect
PROBHD 5 mm CPTCI 1H-
PULPROG zgpg
TD 131072
SOLVENT MeOD
NS 2811
DS 8
SWH 48076.922 Hz
FIDRES 0.366798 Hz
AQ 1.3631488 sec
RG 880.17
DW 10.400 usec
DE 19.73 usec
TE 298.0 K
D1 3.00000000 sec
D11 0.03000000 sec
TD0 6
======== CHANNEL f1 ========
SFO1 201.2129669 MHz
NUC1 13C
P1 11.70 usec
PLW1 100.00000000 W
======== CHANNEL f2 ========
SFO2 800.1325604 MHz
NUC2 1H
CPDPRG[2 waltz64
PCPD2 60.00 usec
PLW2 10.00000000 W
PLW12 0.27778000 W
PLW13 0.13971999 W
F2 - Processing parameters
SI 262144
SF 201.1925353 MHz
WDW EM
SSB 0
LB 0.50 Hz
GB 0
PC 1.20
180 170 160 150 140 130 120 110 100 90 80 70 ppm

S1K

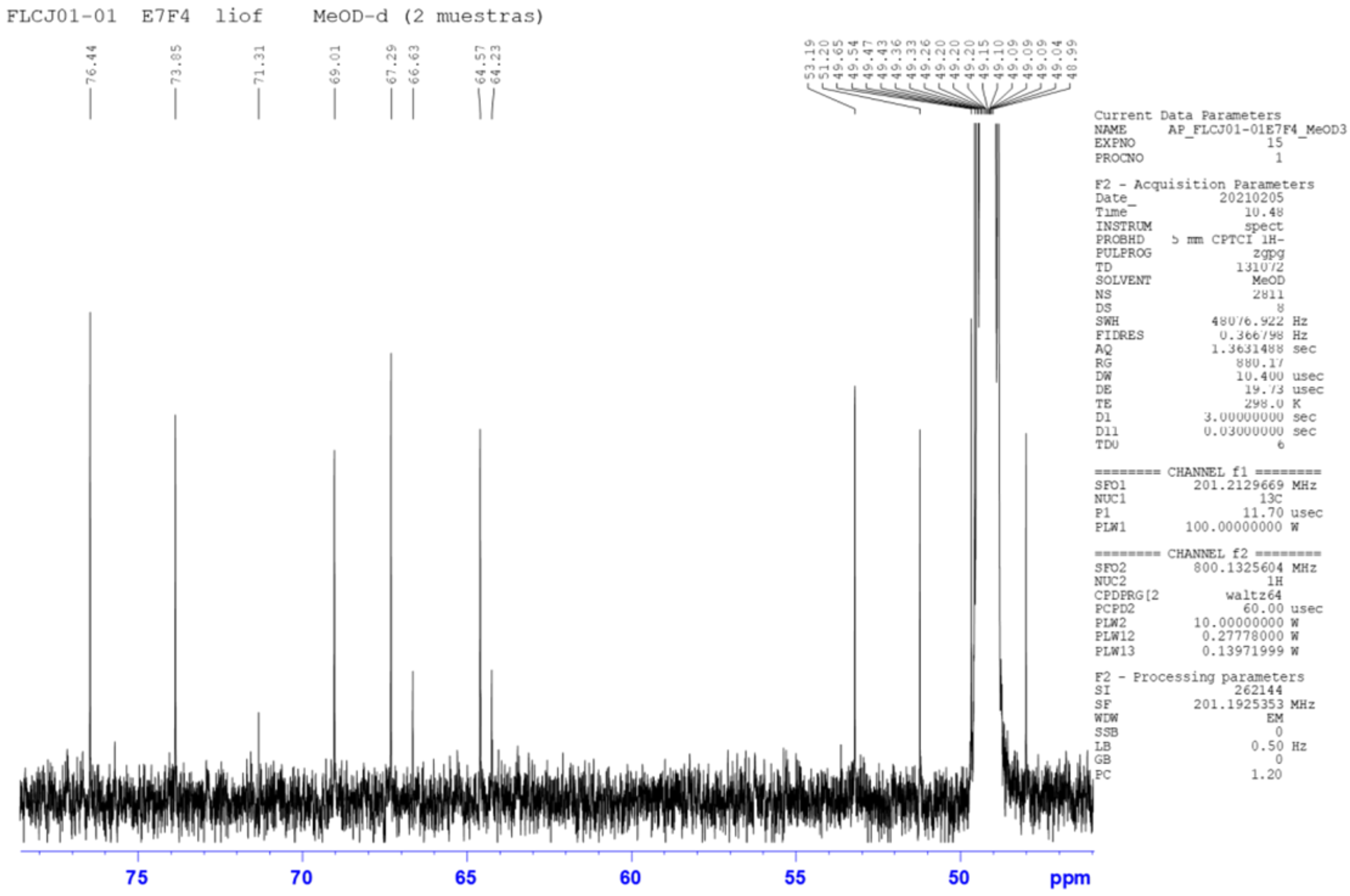
FLCJ01-01 E7F4 liof MeOD-d (2 muestras)
76.44
73.85
71.31
69.01
67.29
66.63
64.57
64.23
53.19
51.20
49.65
49.54
49.47
49.43
49.36
49.33
49.26
49.20
49.20
49.15
49.10
49.09
49.09
49.04
48.99
Current Data Parameters
NAME AP_FLCJ01-01E7F4_MeOD3
EXPNO 15
PROCNO 1
F2 - Acquisition Parameters
Date_ 20210205
Time 10.48
INSTRUM spect
PROBHD 5 mm CPTCI 1H-
PULPROG zgpg
TD 131072
SOLVENT MeOD
NS 2811
DS 8
SWH 48076.922 Hz
FIDRES 0.366798 Hz
AQ 1.3631488 sec
RG 880.17
DW 10.400 usec
DE 19.73 usec
TE 298.0 K
D1 3.00000000 sec
D11 0.03000000 sec
TD0 6
======== CHANNEL f1 ========
SFO1 201.2129669 MHz
NUC1 13C
P1 11.70 usec
PLW1 100.00000000 W
======== CHANNEL f2 ========
SFO2 800.1325604 MHz
NUC2 1H
CPDPRG[2 waltz64
PCPD2 60.00 usec
PLW2 10.00000000 W
PLW12 0.27778000 W
PLW13 0.13971999 W
F2 - Processing parameters
SI 262144
SF 201.1925353 MHz
WDW EM
SSB 0
LB 0.50 Hz
GB 0
PC 1.20
75
70
65
60
55
50
ppm

S1L

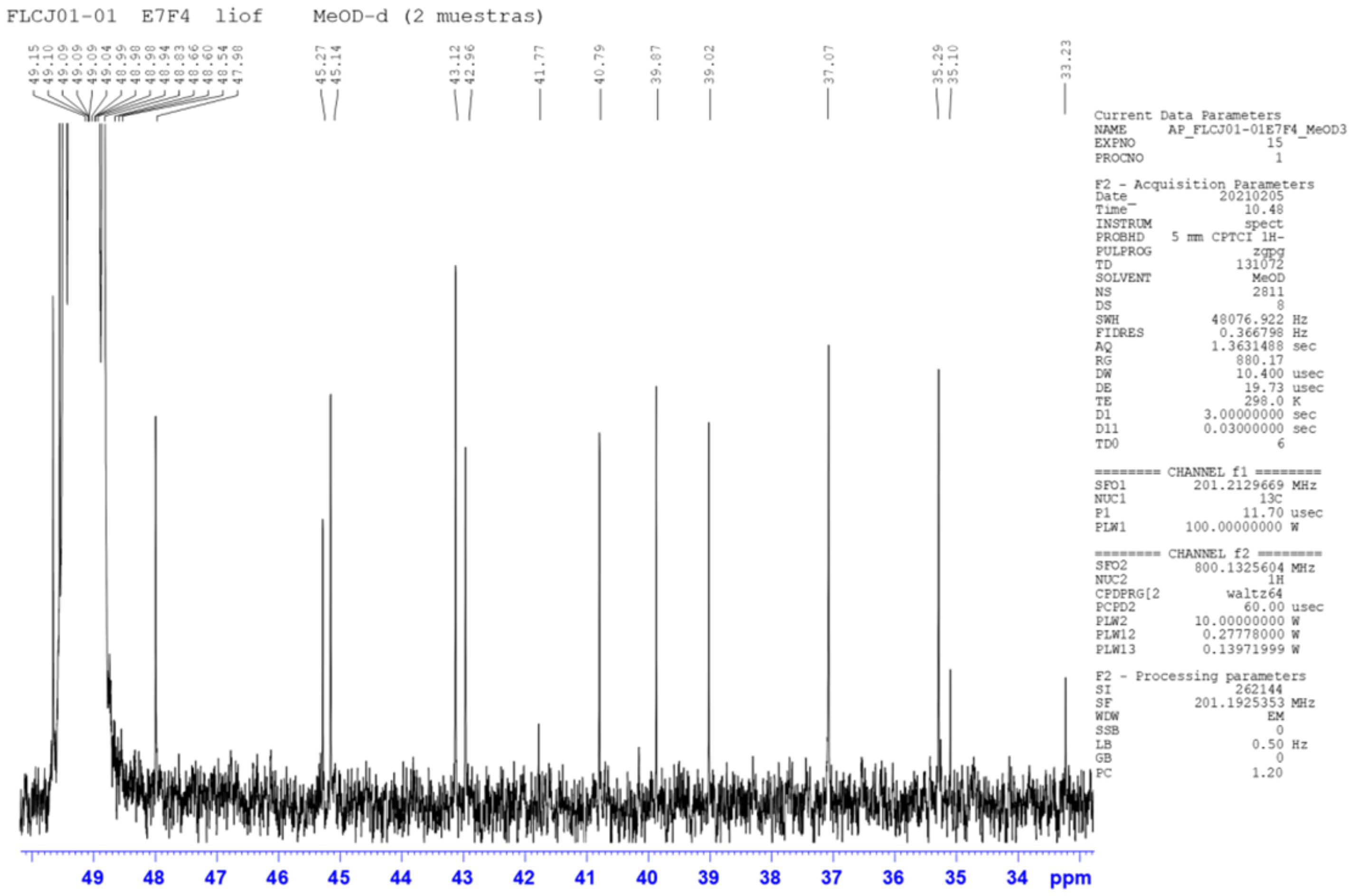
FLCJ01-01 E7F4 liof MeOD-d (2 muestras)
49.15
49.10
49.09
49.09
49.09
49.04
48.99
48.98
48.98
48.94
48.83
48.66
48.60
48.54
47.98
45.27
45.14
43.12
42.96
41.77
40.79
39.87
39.02
37.07
35.29
35.10
33.23
Current Data Parameters
NAME AP_FLCJ01-01E7F4_MeOD3
EXPNO 15
PROCNO 1
F2 - Acquisition Parameters
Date_ 20210205
Time_ 10.48
INSTRUM spect
PROBHD 5 mm CPTCI 1H-
PULPROG zgpg
TD 131072
SOLVENT MeOD
NS 2811
DS 8
SWH 48076.922 Hz
FIDRES 0.366798 Hz
AQ 1.3631488 sec
RG 880.17
DW 10.400 usec
DE 19.73 usec
TE 298.0 K
D1 3.00000000 sec
D11 0.03000000 sec
TD0 6
======== CHANNEL f1 ========
SFO1 201.2129669 MHz
NUC1 13C
P1 11.70 usec
PLW1 100.00000000 W
======== CHANNEL f2 ========
SFO2 800.1325604 MHz
NUC2 1H
CPDPRG[2 waltz64
PCPD2 60.00 usec
PLW2 10.00000000 W
PLW12 0.27778000 W
PLW13 0.13971999 W
F2 - Processing parameters
SI 262144
SF 201.1925353 MHz
WDW EM
SSB 0
LB 0.50 Hz
GB 0
PC 1.20
49 48 47 46 45 44 43 42 41 40 39 38 37 36 35 34 ppm

S1M

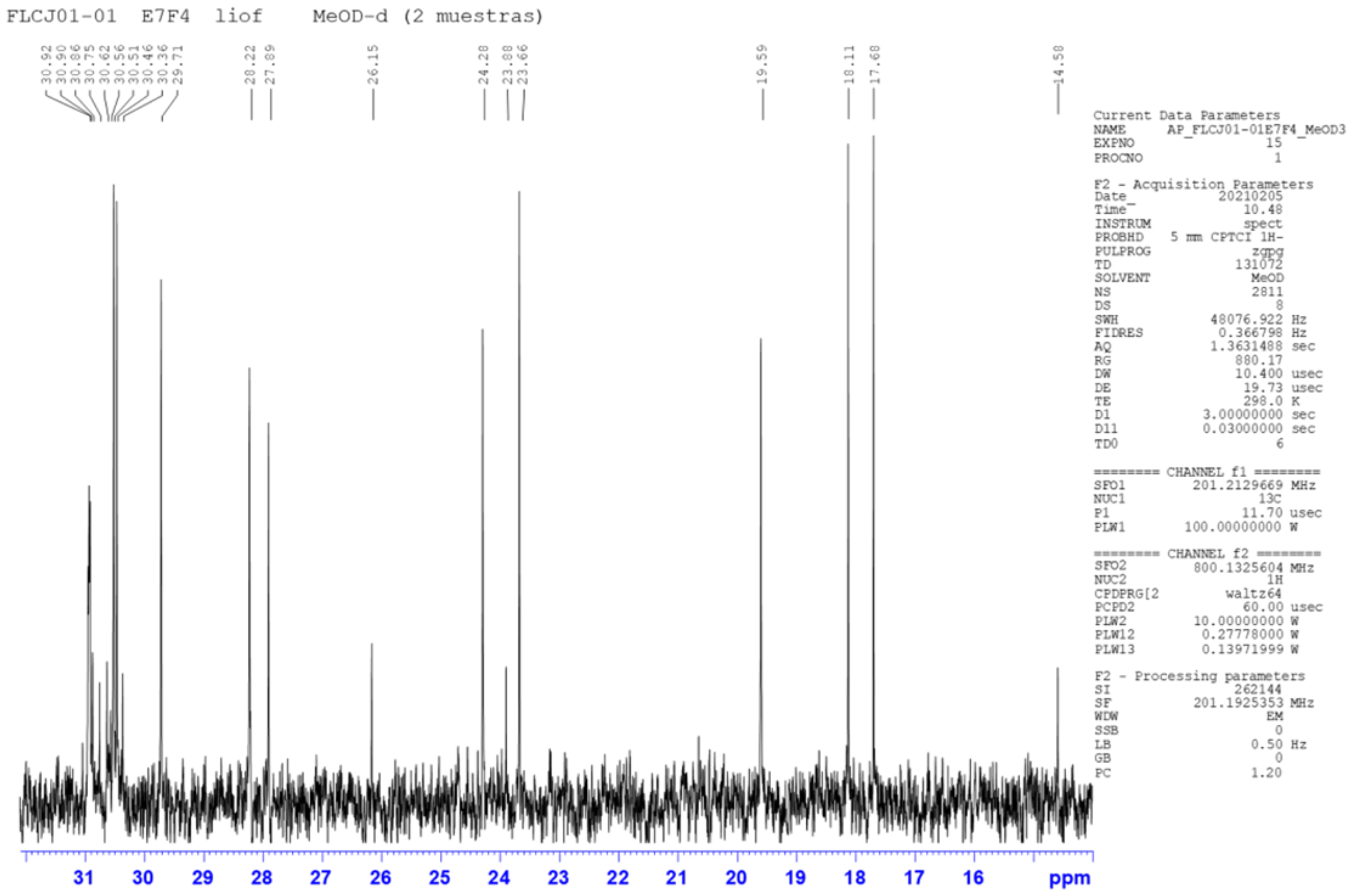
FLCJ01-01 E7F4 liof MeOD-d (2 muestras)
30.92 30.90 30.86 30.75 30.62 30.56 30.51 30.46 30.36 29.71
28.22 27.89
26.15
24.28 23.88 23.66
19.59
18.11 17.68
14.58
Current Data Parameters
NAME AP_FLCJ01-01E7F4_MeOD3
EXPNO 15
PROCNO 1
F2 - Acquisition Parameters
Date_ 20210205
Time 10.48
INSTRUM spect
PROBHD 5 mm CPTCI 1H-
PULPROG zgpg
TD 131072
SOLVENT MeOD
NS 2811
DS 8
SWH 48076.922 Hz
FIDRES 0.366798 Hz
AQ 1.3631488 sec
RG 880.17
DW 10.400 usec
DE 19.73 usec
TE 298.0 K
D1 3.00000000 sec
D11 0.03000000 sec
TD0 6
======== CHANNEL f1 ========
SFO1 201.2129669 MHz
NUC1 13C
P1 11.70 usec
PLW1 100.00000000 W
======== CHANNEL f2 ========
SFO2 800.1325604 MHz
NUC2 1H
CPDPRG[2 waltz64
PCPD2 60.00 usec
PLW2 10.00000000 W
PLW12 0.27778000 W
PLW13 0.13971999 W
F2 - Processing parameters
SI 262144
SF 201.1925353 MHz
WDW EM
SSB 0
LB 0.50 Hz
GB 0
PC 1.20
31 30 29 28 27 26 25 24 23 22 21 20 19 18 17 16 ppm

Figure S2: NMR spectra of E5-E6 sample (phlomispurtetraolone). S2A and S2B: ^{1}H, S2C: COSY, S2D: TOCSY, S2E: NOESY, S2F to S2H: HSQC, S2I: HSQC-TOCSY, S2J and S2K: HMBC.

S2A

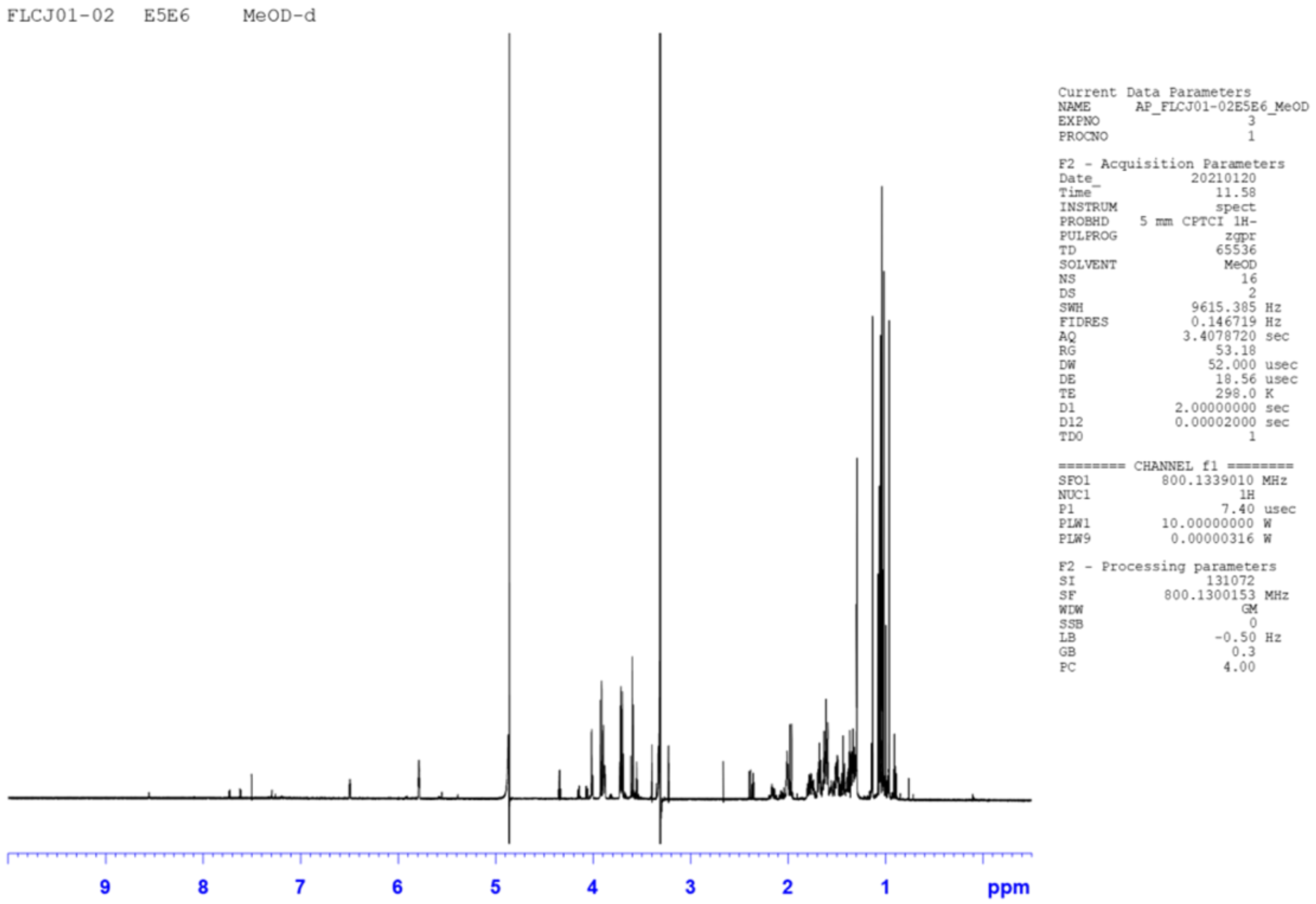

S2B

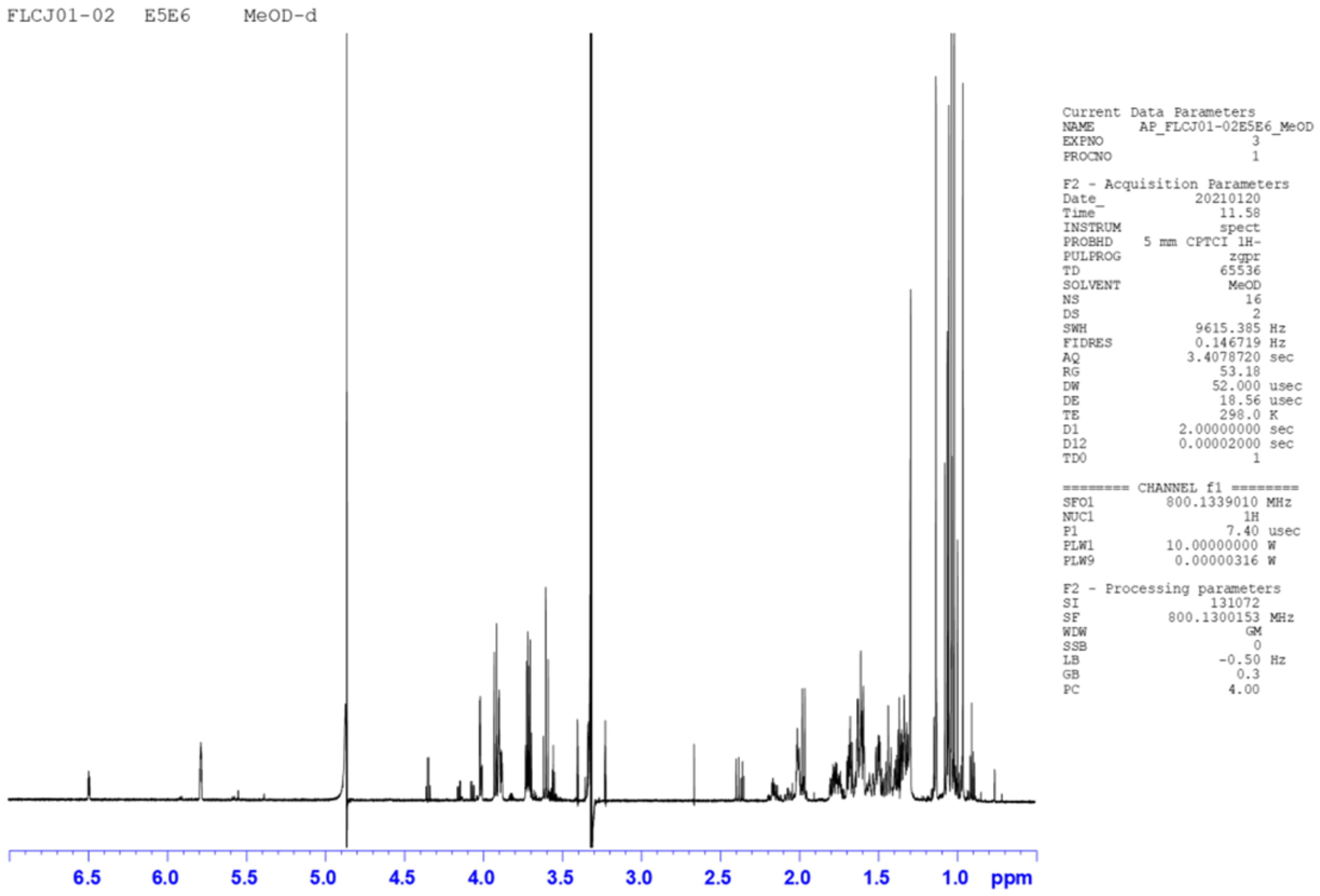
FLCJ01-02 E5E6 MeOD-d
6.5
6.0
5.5
5.0
4.5
4.0
3.5
3.0
2.5
2.0
1.5
1.0
ppm
Current Data Parameters
NAME AP_FLCJ01-02E5E6_MeOD
EXPNO 3
PROCNO 1
F2 - Acquisition Parameters
Date_ 20210120
Time 11.58
INSTRUM spect
PROBHD 5 mm CPTCI 1H-
PULPROG zgpr
TD 65536
SOLVENT MeOD
NS 16
DS 2
SWH 9615.385 Hz
FIDRES 0.146719 Hz
AQ 3.4078720 sec
RG 53.18
DW 52.000 usec
DE 18.56 usec
TE 298.0 K
D1 2.00000000 sec
D12 0.00002000 sec
TD0 1
======== CHANNEL f1 ========
SFO1 800.1339010 MHz
NUC1 1H
P1 7.40 usec
PLW1 10.00000000 W
PLW9 0.00000316 W
F2 - Processing parameters
SI 131072
SF 800.1300153 MHz
WDW GM
SSB 0
LB -0.50 Hz
GB 0.3
PC 4.00

S2C

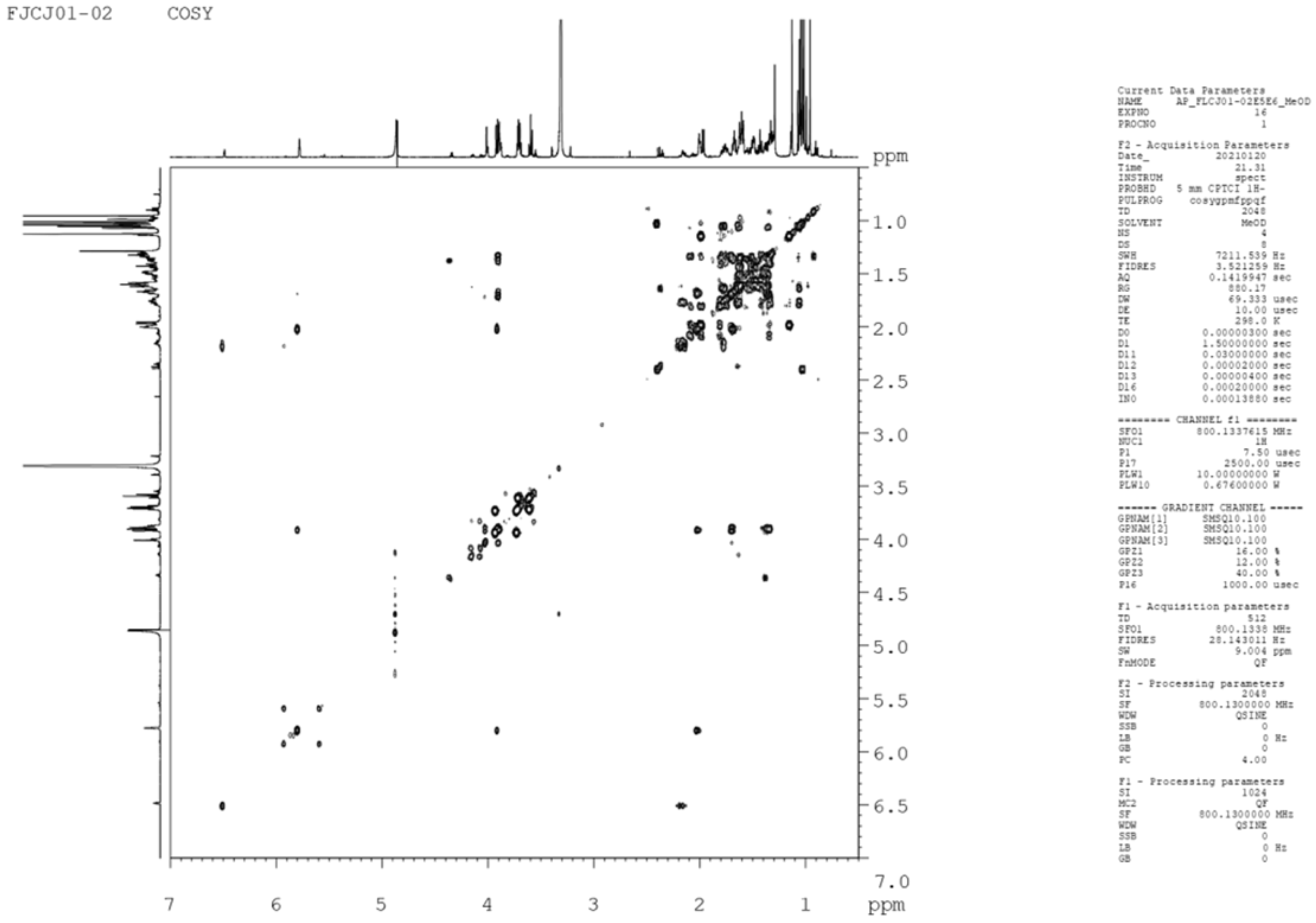
FJCJ01-02 COSY
ppm
1.0
1.5
2.0
2.5
3.0
3.5
4.0
4.5
5.0
5.5
6.0
6.5
7.0
7
6
5
4
3
2
1
ppm
Current Data Parameters
NAME AP_FLCJ01-02E5E6_MeOD
EXPNO 16
PROCNO 1
F2 - Acquisition Parameters
Date_ 20210120
Time 21.31
INSTRUM spect
PROBHD 5 mm CPTCI 1H-
PULPROG cosygpmfppqf
TD 2048
SOLVENT MeOD
NS 4
DS 8
SWH 7211.539 Hz
FIDRES 3.521259 Hz
AQ 0.1419947 sec
RG 880.17
DW 69.333 usec
DE 10.00 usec
TE 298.0 K
D0 0.00000300 sec
D1 1.50000000 sec
D11 0.03000000 sec
D12 0.00002000 sec
D13 0.00000400 sec
D16 0.00020000 sec
IN0 0.00013880 sec
======== CHANNEL f1 ========
SFO1 800.1337615 MHz
NUC1 1H
P1 7.50 usec
P17 2500.00 usec
PLW1 10.00000000 W
PLW10 0.67600000 W
====== GRADIENT CHANNEL =====
GPNAM[1] SMSQ10.100
GPNAM[2] SMSQ10.100
GPNAM[3] SMSQ10.100
GPZ1 16.00 %
GPZ2 12.00 %
GPZ3 40.00 %
P16 1000.00 usec
F1 - Acquisition parameters
TD 512
SFO1 800.1338 MHz
FIDRES 28.143011 Hz
SW 9.004 ppm
FnMODE QF
F2 - Processing parameters
SI 2048
SF 800.1300000 MHz
WDW QSINE
SSB 0
LB 0 Hz
GB 0
PC 4.00
F1 - Processing parameters
SI 1024
MC2 QF
SF 800.1300000 MHz
WDW QSINE
SSB 0
LB 0 Hz
GB 0

S2D

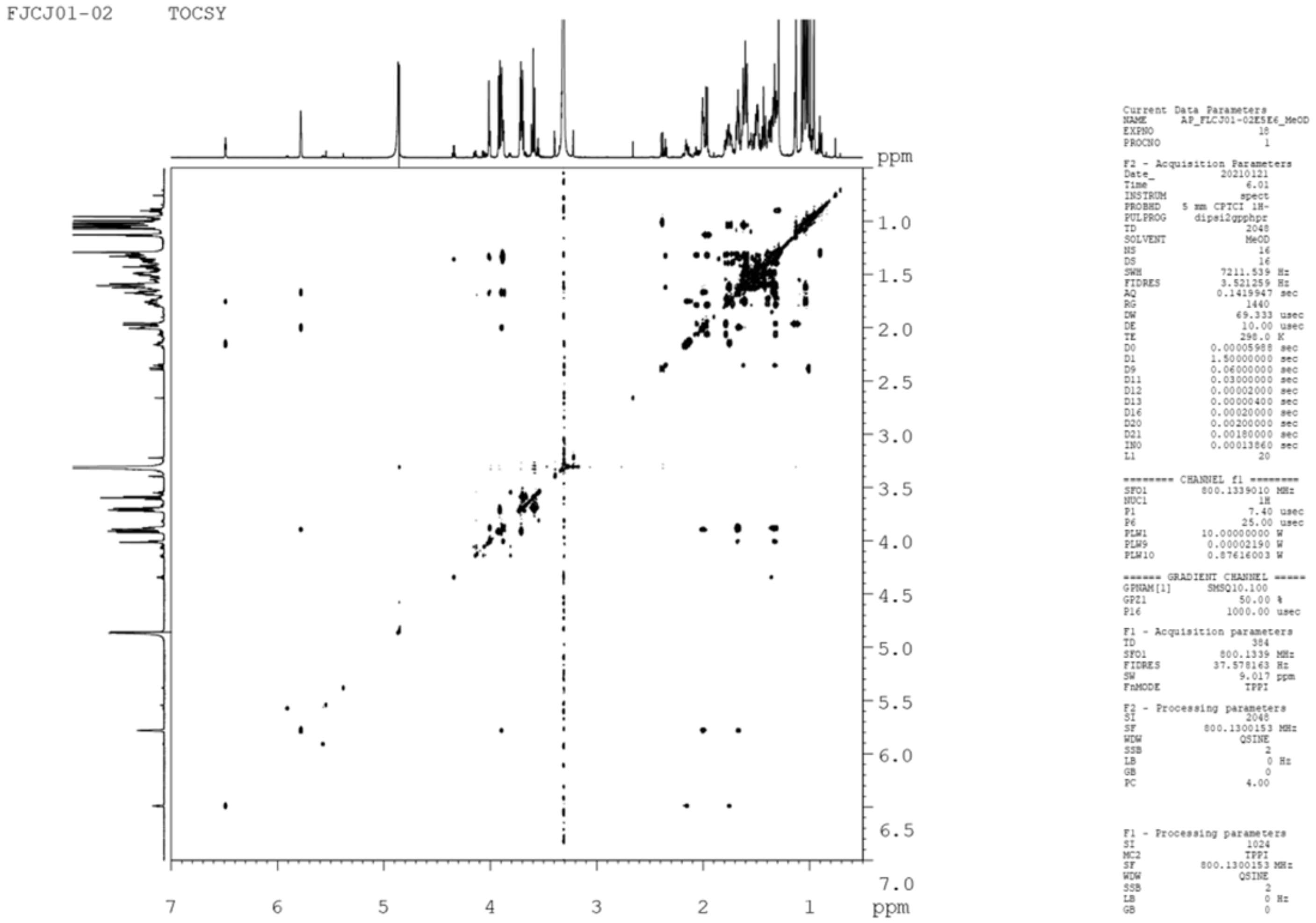
FJCJ01-02 TOCSY
ppm
1.0
1.5
2.0
2.5
3.0
3.5
4.0
4.5
5.0
5.5
6.0
6.5
7.0
7
6
5
4
3
2
1
ppm
Current Data Parameters
NAME AP_FLCJ01-02ESE6_MeOD
EXPNO 18
PROCNO 1
F2 - Acquisition Parameters
Date_ 20210121
Time 6.01
INSTRUM spect
PROBHD 5 mm CPTCI 1H-
PULPROG dipsi2gpphpr
TD 2048
SOLVENT MeOD
NS 16
DS 16
SWH 7211.539 Hz
FIDRES 3.521259 Hz
AQ 0.1419947 sec
RG 1440
DW 69.333 usec
DE 10.00 usec
TE 298.0 K
D0 0.00005988 sec
D1 1.50000000 sec
D9 0.06000000 sec
D11 0.03000000 sec
D12 0.00002000 sec
D13 0.00000400 sec
D16 0.00020000 sec
D20 0.00200000 sec
D21 0.00180000 sec
IN0 0.00013860 sec
L1 20
======== CHANNEL f1 ========
SFO1 800.1339010 MHz
NUC1 1H
P1 7.40 usec
P6 25.00 usec
PLW1 10.00000000 W
PLW9 0.00002190 W
PLW10 0.87616003 W
====== GRADIENT CHANNEL =====
GPNAM[1] SMSQ10.100
GPZ1 50.00 %
P16 1000.00 usec
F1 - Acquisition parameters
TD 384
SFO1 800.1339 MHz
FIDRES 37.578163 Hz
SW 9.017 ppm
FnMODE TPPI
F2 - Processing parameters
SI 2048
SF 800.1300153 MHz
WDW QSINE
SSB 2
LB 0 Hz
GB 0
PC 4.00
F1 - Processing parameters
SI 1024
MC2 TPPI
SF 800.1300153 MHz
WDW QSINE
SSB 2
LB 0 Hz
GB 0

S2E

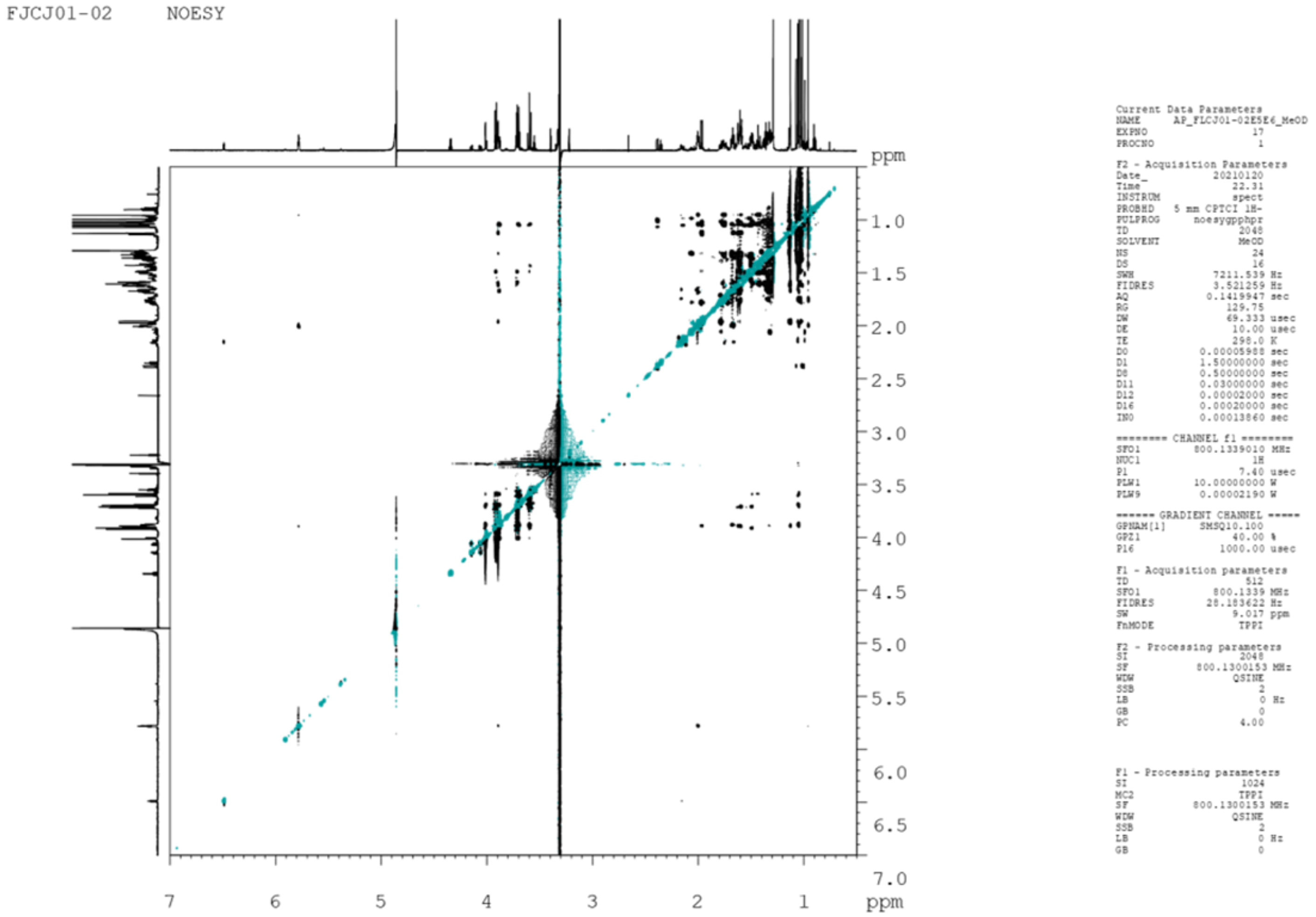
FJCJ01-02 NOESY
ppm
1.0
1.5
2.0
2.5
3.0
3.5
4.0
4.5
5.0
5.5
6.0
6.5
7.0
7 6 5 4 3 2 1
ppm
Current Data Parameters
NAME AP_FLCJ01-02E5E6_MeOD
EXPNO 17
PROCNO 1
F2 - Acquisition Parameters
Date_ 20210120
Time 22.31
INSTRUM spect
PROBHD 5 mm CPTCI 1H-
PULPROG noesygpphpr
TD 2048
SOLVENT MeOD
NS 24
DS 16
SWH 7211.539 Hz
FIDRES 3.521259 Hz
AQ 0.1419947 sec
RG 129.75
DW 69.333 usec
DE 10.00 usec
TE 298.0 K
D0 0.00005988 sec
D1 1.50000000 sec
D8 0.50000000 sec
D11 0.03000000 sec
D12 0.00002000 sec
D16 0.00020000 sec
IN0 0.00013860 sec
======== CHANNEL f1 ========
SFO1 800.1339010 MHz
NUC1 1H
P1 7.40 usec
PLW1 10.00000000 W
PLW9 0.00002190 W
====== GRADIENT CHANNEL =====
GPNAM[1] SMSQ10.100
GPZ1 40.00 %
P16 1000.00 usec
F1 - Acquisition parameters
TD 512
SFO1 800.1339 MHz
FIDRES 28.183622 Hz
SW 9.017 ppm
FnMODE TPPI
F2 - Processing parameters
SI 2048
SF 800.1300153 MHz
WDW QSINE
SSB 2
LB 0 Hz
GB 0
PC 4.00
F1 - Processing parameters
SI 1024
MC2 TPPI
SF 800.1300153 MHz
WDW QSINE
SSB 2
LB 0 Hz
GB 0

S2F

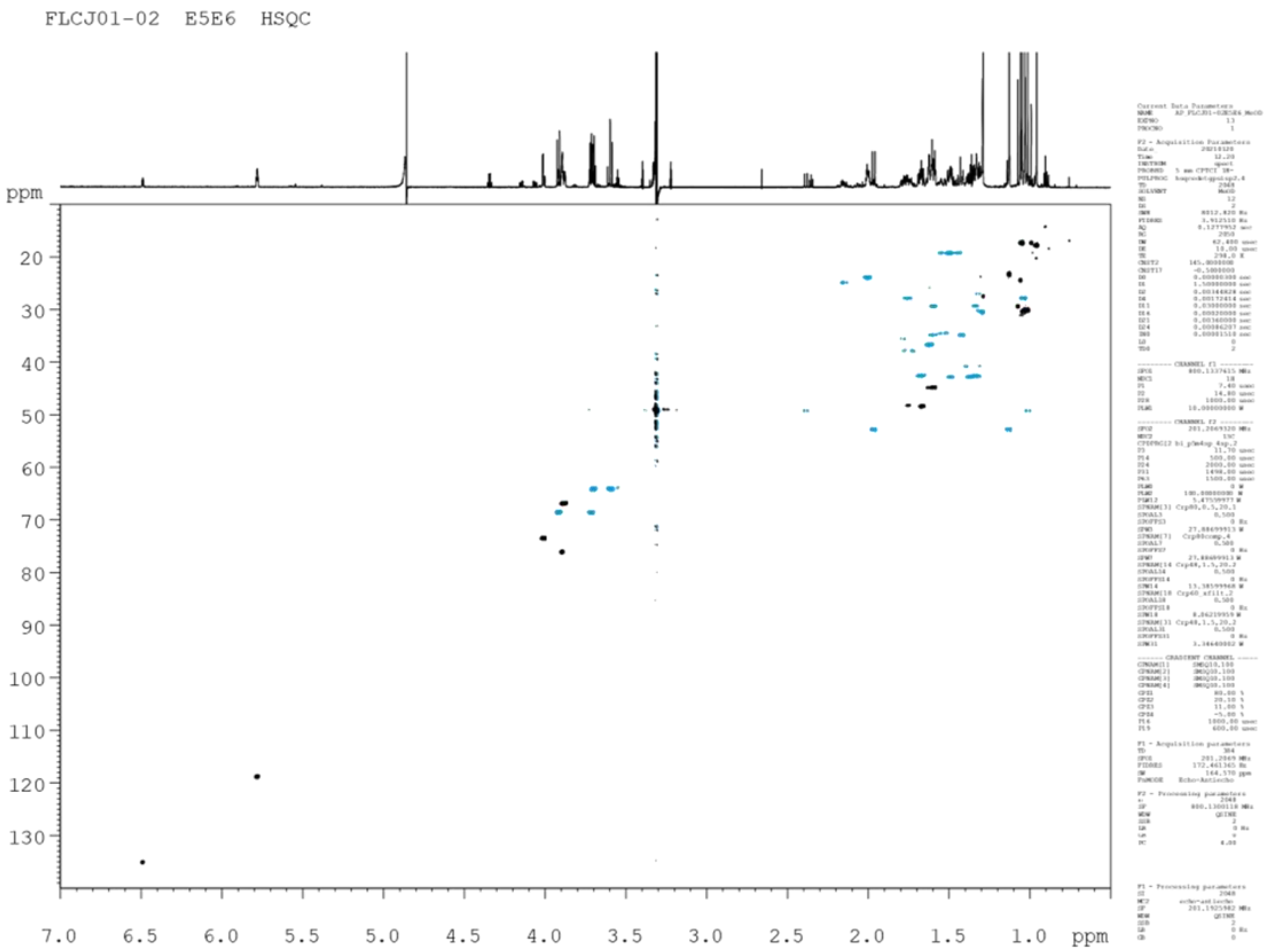
FLCJ01-02 E5E6 HSQC
ppm
20
30
40
50
60
70
80
90
100
110
120
130
7.0
6.5
6.0
5.5
5.0
4.5
4.0
3.5
3.0
2.5
2.0
1.5
1.0
ppm

S2G

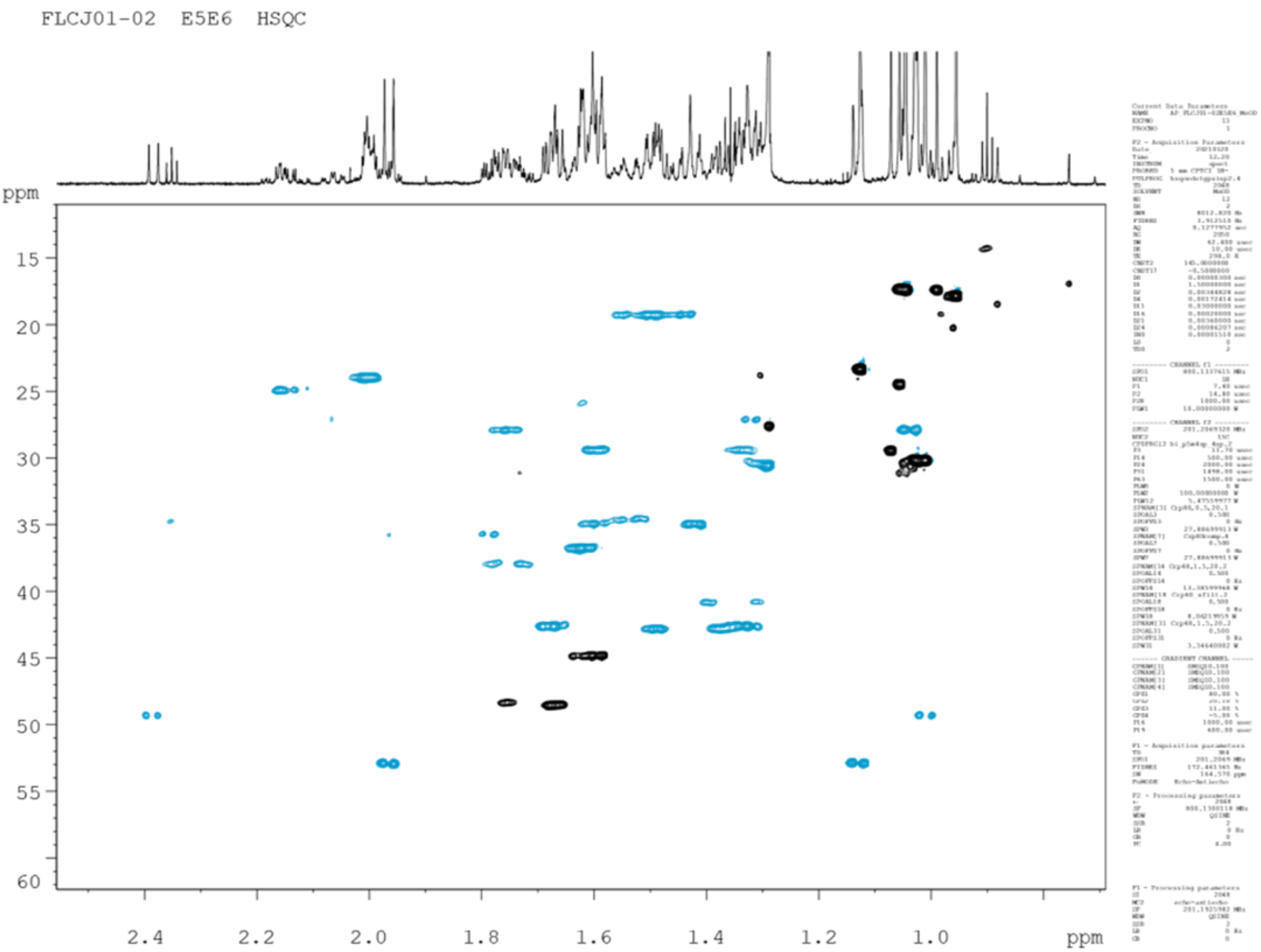

FLCJ01-02 E5E6 HSQC
ppm
15
20
25
30
35
40
45
50
55
60
2.4
2.2
2.0
1.8
1.6
1.4
1.2
1.0
ppm

S2H

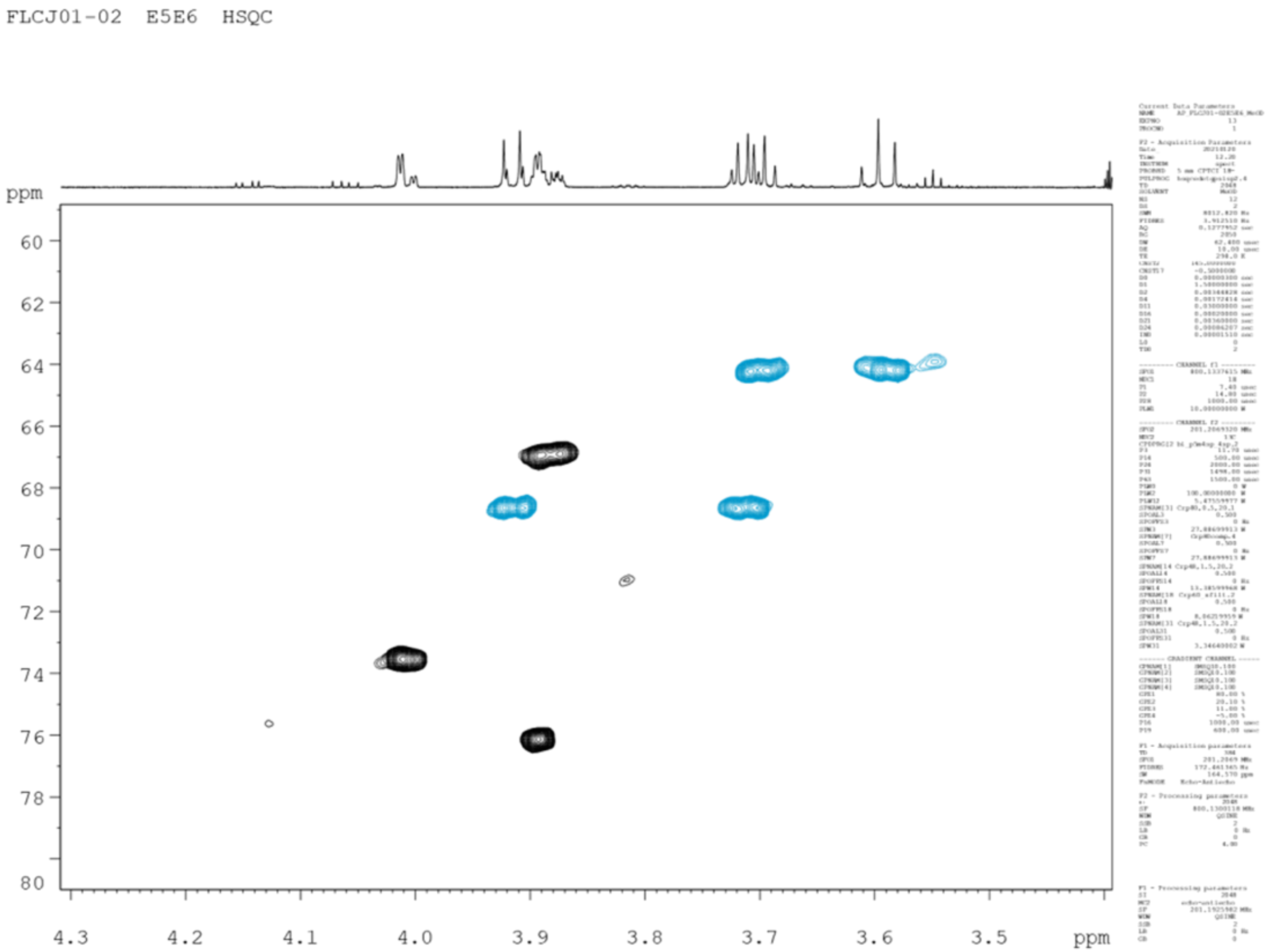

S2I

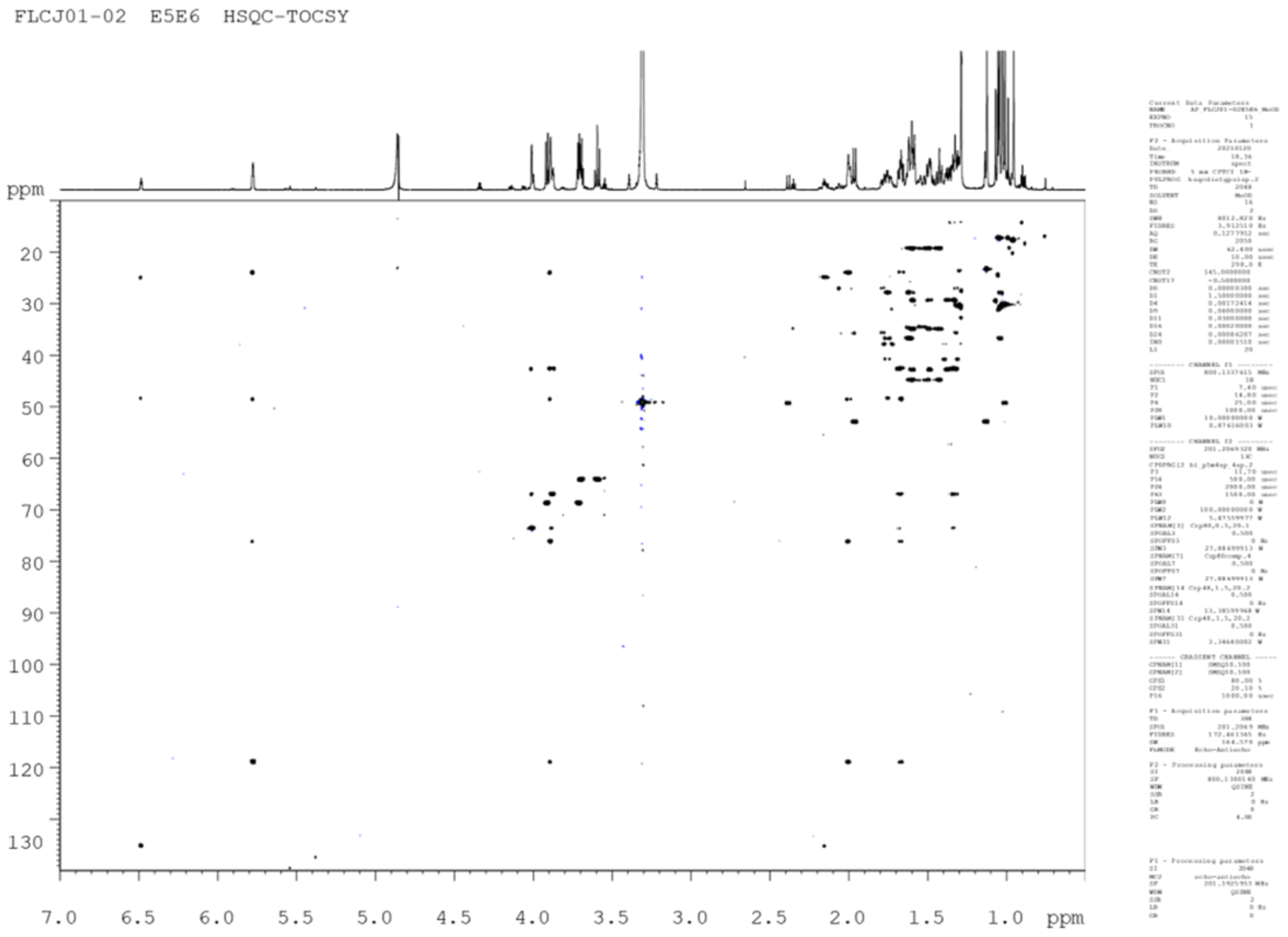
FLCJ01-02 E5E6 HSQC-TOCSY
ppm
20
30
40
50
60
70
80
90
100
110
120
130
7.0
6.5
6.0
5.5
5.0
4.5
4.0
3.5
3.0
2.5
2.0
1.5
1.0
ppm

S2J

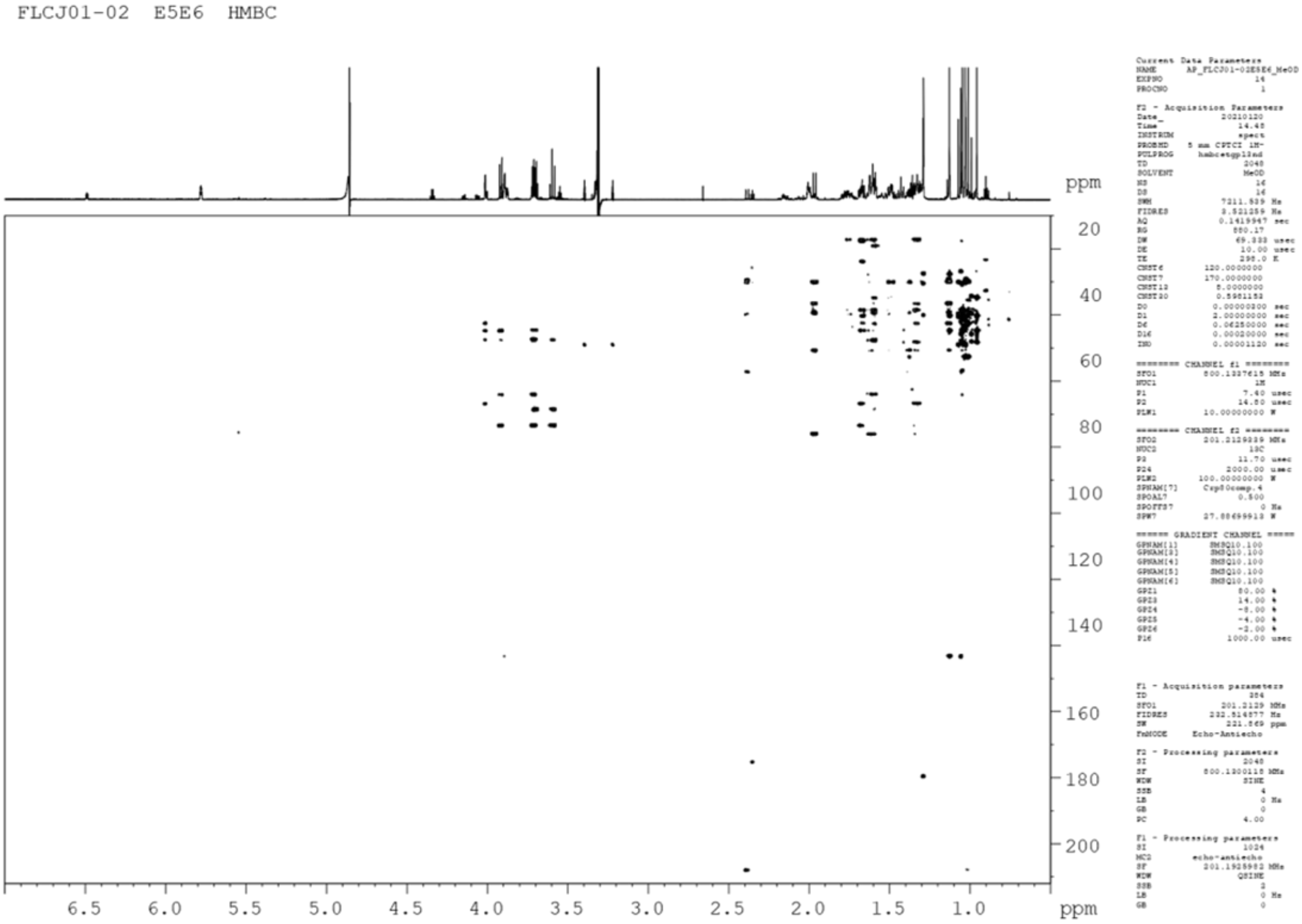

S2K

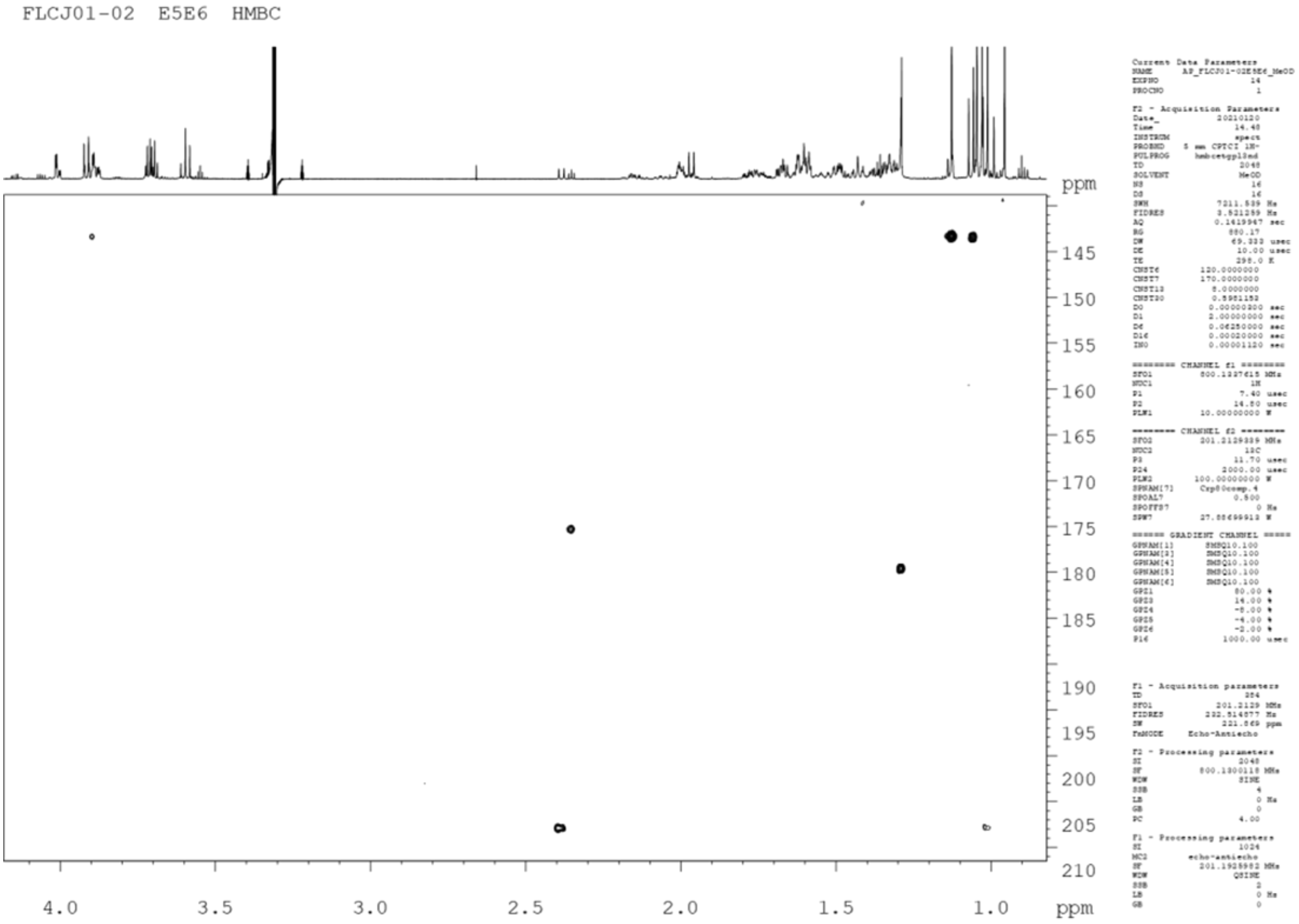

FLCJ01-02 E5E6 HMBC
ppm
145
150
155
160
165
170
175
180
185
190
195
200
205
210
4.0
3.5
3.0
2.5
2.0
1.5
1.0
ppm

Figure S3: Comparison of E7-F4 (red, phlomispentaol A) and E5-E6 (blue, phlomispurtetraolone) NMR spectra. The marked signals correspond to phlomispurtetraolone. S3A: Superposition of ^{1}H spectra for both samples. S3B: Superposition of ^{1}H spectra for both samples, with magnification of the olefinic zone (C=CH) (a), the CH and CH2 groups bound to O zone (b), the partial aliphatic zone (c), and the methyl groups aliphatic zone (d). S3C: Superposition of the ^{1}H-^{13}C-HSQC edited spectra (E7-F4 (CH_2 pink, CH/CH_3 red) and E5-E6 (CH_2 green, CH/CH_3 black).

S3A

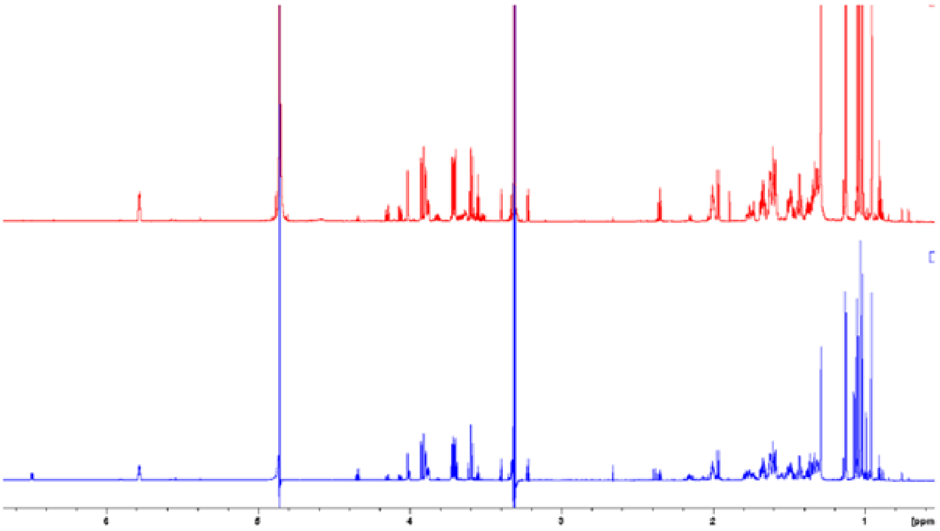

S3B

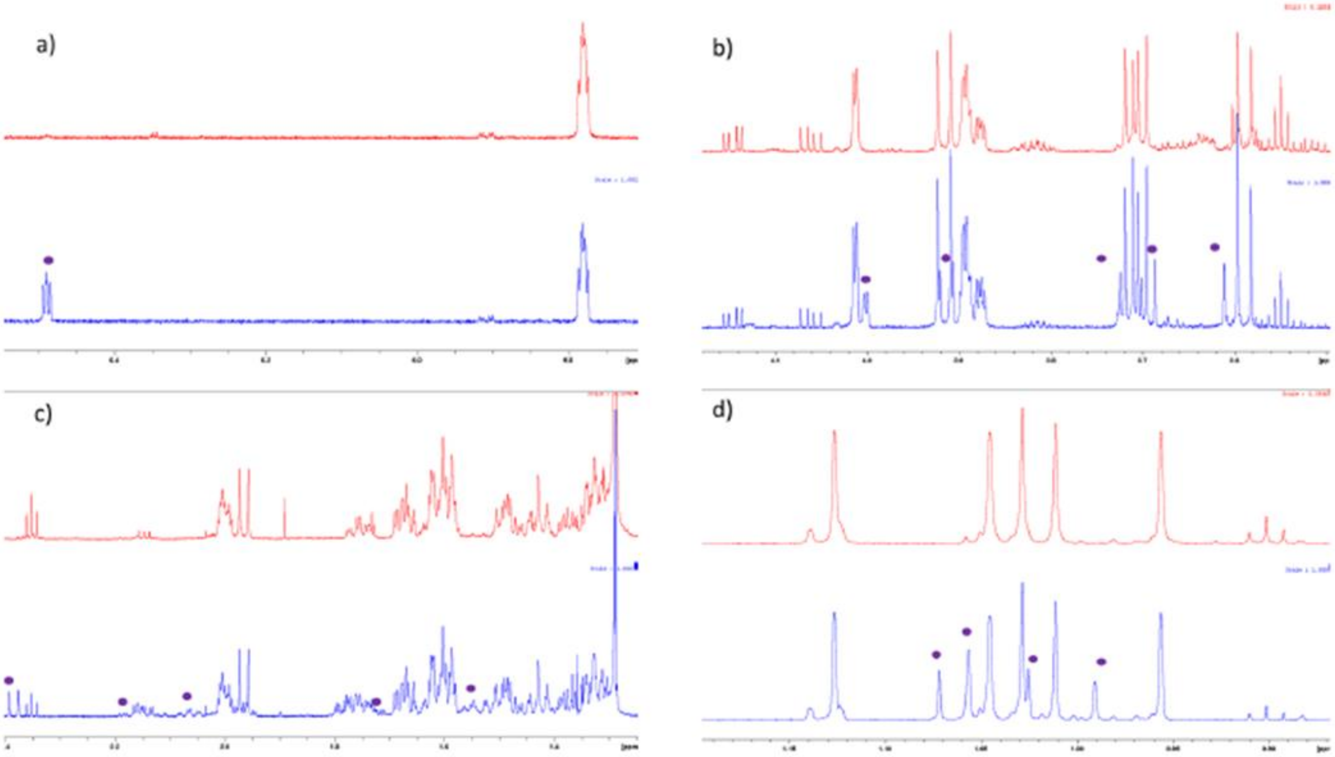

S3C

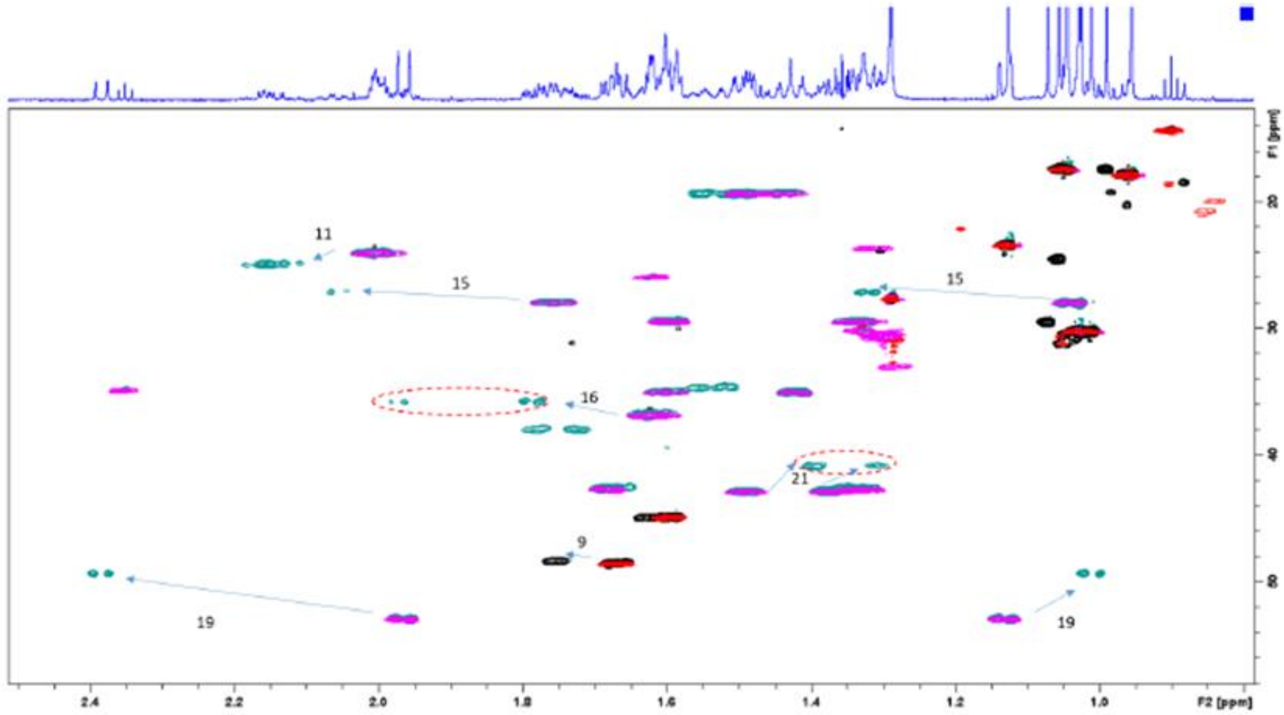

Supplement: Supplementary file 1 [file DataSheet_1.pdf]
